# Bibliometric Review of the Step Test: A Comprehensive Analysis of Research Trends and Development

**DOI:** 10.1186/s40798-024-00764-y

**Published:** 2024-08-28

**Authors:** Tatiana Sampaio, Daniel A. Marinho, José A. Bragada, Jorge E. Morais

**Affiliations:** 1 Department of Sports Sciences, Instituto Politécnico de Bragança, Campus Sta. Apolónia, Apartado 1101, 5301-856 Bragança, Portugal; 2 Research Center in Sports, Health and Human Development (CIDESD), Covilhã, Portugal; 3 Department of Sport Sciences, University of Beira Interior, Covilhã, Portugal; 4 Research Centre for Active Living and Wellbeing (LiveWell), Instituto Politécnico de Bragança, Bragança, Portugal

**Keywords:** Bibliometric analysis, Cardiorespiratory fitness, Timed step tests, Historical development

## Abstract

**Background:**

The step test provides valuable information on cardiorespiratory parameters such as maximal oxygen uptake and heart rate. Cardiorespiratory capacity is critical to health-related fitness, with heart rate recovery after exercise serving as a predictor of mortality risk.

**Main Body:**

The primary objective of this review was to identify trends, key contributors, and emerging themes in the step test literature through 2023 using the Web of Science Core Collection. Bibliometric data extraction and analysis were performed using a dedicated bibliometric software (VOSviewer). The analysis showed continued interest through 2021. The research categories highlight the multidisciplinary nature of the step test, covering cardiovascular systems, respiratory systems, sports sciences, and medicine. It has gained global attention, with 31 countries involved, with Brazil and the United States leading the way. The diversity of contributing nations is increasing, reflecting a growing global interest. With 111 journals involved, such as Respiratory Care and Medicine and Science in Sports and Exercise, step test research is spread across the academic landscape. With 761 contributing researchers, there is a collaborative and diverse community. The top 10 institutions, including the University of Alabama System and Monash University, illustrate the variety of settings in which step test studies are conducted. Step test studies span multiple disciplines, underscoring their adaptability. The clusters identified in this bibliometric analysis of the step test literature guide future research, suggesting avenues for refining protocols, exploring health implications, optimizing tests for specific conditions such as chronic obstructive pulmonary disease, and adapting step tests in diverse populations.

**Conclusions:**

Practical implications highlight the role of the step test in cardiovascular risk assessment, fitness monitoring, and rehabilitation. This broad review underscores the relevance of the step test in diverse settings, reflecting its adaptability and ease of application across occupational and clinical settings.

**Supplementary Information:**

The online version contains supplementary material available at 10.1186/s40798-024-00764-y.

## Key Points

Global interest and research in the step test has grown significantly, with contributions from 31 countries and diverse disciplines, highlighting the adaptability and multidisciplinary relevance of the step test.

Practical implications of the step test include its critical role in cardiovascular risk assessment, fitness monitoring, and rehabilitation.

**Supplementary Information:**

The online version contains supplementary material available at 10.1186/s40798-024-00764-y.

## Background

Cardiorespiratory fitness, which encompasses the ability of the circulatory, respiratory, and muscular systems to deliver oxygen during prolonged physical exercise, is a fundamental pillar of health-related physical fitness [1]. A substantial body of research indicates that a variety of cardiovascular and metabolic risk factors, including those related to poor cardiorespiratory fitness, are associated with an increased risk of morbidity and mortality in both men and women [2]. Therefore, it may be suggested that maintaining and assessing cardiorespiratory fitness is essential to prevent losses in aerobic capacity and reduce the associated health risks.

Traditionally, the assessment of cardiorespiratory fitness has relied on metrics such as metabolic equivalent of task (METs) or maximal oxygen consumption (VO_2max_), often assessed by exercise testing on dedicated equipment such as treadmills or bicycle ergometers [3–5]. However, the lack of such equipment outside of laboratory settings has led to the development of alternative, yet reliable, methods for assessing cardiorespiratory fitness. In response to this need, several timed-step tests have been developed to provide practical alternatives for assessing cardiorespiratory fitness. Examples include the Queen's College Step Test [6], the YMCA Step Test [7] and the StepTest4all [8, 9], among others, which offer opportunities for widespread fitness assessment without the constraints of laboratory equipment.

Step tests are an accessible, easy-to-use, portable, and sustainable method for estimating submaximal VO_2max_ [10]. They have great potential for use in assessing the health of adult populations, both in a rehabilitation context and as a safe and useful method of measuring cardiorespiratory fitness under submaximal conditions.

Research has shown that exercise test data can be highly predictive, especially for functional capacity and exercise heart rate dynamics such as heart rate recovery (HRR) [11, 12]. HRR is defined as the reduction in heart rate from peak exercise during a stress test to the rate one minute after exercise is stopped [13]. Analysis of post-exercise cardiac autonomic recovery provides valuable insights into autonomic nervous system activity, with slow HRR indicating cardiac autonomic dysfunction, a robust predictor of cardiovascular morbidity and mortality [14], particularly a delayed decline in heart rate during the first minute after graded exercise, reflecting reduced vagal activity [13]. Improved aerobic fitness positively influences autonomic control of post-exercise heart rate and preserves vagal re-entry velocity in healthy middle-aged adults [15]. Therefore, maintaining good cardiorespiratory fitness through testing is critical to preventing health decline in the general population [16].

By bridging the gap between laboratory-based assessments and real-world scenarios, these step tests have practical implications that extend beyond the research lab. The step tests have profound practical implications because they serve as sensitive and accurate indicators of an individual's regular physical activity level and overall cardiorespiratory fitness [17]. In addition, they provide a simple and accessible means of fitness assessment, eliminating the need for specialized equipment or expert supervision [8]. Whether used in home-based fitness monitoring [18], clinical evaluations [19], or research studies involving diverse populations [20], the step test has transcended its simplicity to become an indispensable tool for health and exercise professionals.

The literature on the step test includes systematic reviews in different populations [17, 21, 22]. For example, the 2016 systematic review by Bennett et al. [17] aimed to assess the validity and reliability of submaximal step test protocols as a method to estimate VO_2max_ in healthy adults (18–65 years) against a validated measure of VO_2max_. The authors highlighted the significant correlation between VO_2max_ and overall health, emphasizing its relevance in health assessment for both the general adult population and in rehabilitation settings. This correlation provides the basis for safe and effective health monitoring. Proactive testing and maintenance of cardiorespiratory fitness is seen as a potential safeguard against declines in general health. In particular, the step test is proving to be a user-friendly and ecologically valid approach to assessing submaximal cardiorespiratory fitness in a variety of settings, thereby enhancing its practicality in health assessment. Nevertheless, to the best of our knowledge, a notable research gap on this topic remains in the form of a comprehensive bibliometric review. Unlike systematic reviews that focus on the clinical effectiveness of the step test, a bibliometric review provides a unique perspective by systematically analyzing publication trends, key contributors, and emerging clusters in the step test literature [23]. Applying a bibliometric review to this topic (i.e., step test) will provide valuable insights into the historical development and current state of step test research through 2023. As mentioned above, this is one of the oldest tests used to measure the cardiorespiratory capacity. Therefore, the primary objective of this study is to conduct a comprehensive bibliometric review of the step test literature, aiming to elucidate its historical development, key contributors, and emerging trends through 2023.

## Main Text

### Data Source and Search Strategy

Web of Science (WoS) is a collection of legitimate worldwide citation databases covering all regions and more than 250 subject areas [24]. Therefore, this literature review relied on the use of the Web of Science Core Collection (WoS by Clarivate Analytics) database. The search period included studies through 2023 (access date: September 19, 2023). The search term used in WoS was “step-test” or “step test” in the title and in the abstract. Using this search technique, the WoS database yielded all published articles containing the word “step test” in the title or in the abstract. The search was performed for articles published up to the year 2023. Any disagreements regarding the selection criteria were discussed and resolved with the assistance of an external referee with expertise in a similar field of research.

### Inclusion Criteria and Exclusion Criteria

The following inclusion criteria were applied: (1) written in English or having the abstract written in English; (2) focused on the step test as a primary topic or assessment method, specifically for evaluating cardiorespiratory capacity; (3) all document types. The exclusion criteria comprised articles that did not meet the following specifications: (1) written in languages other than English with no English abstract or English version provided by the journal; (2) lacked a primary focus on the step test as a topic or assessment method.

### Screening Process

Two authors independently reviewed the titles and abstracts of the identified articles. In cases of doubt about the eligibility of an article, the full text was obtained. Two authors independently assessed each article in two stages: the title, the abstract, and then, the full text of the article. Conflicts over eligibility were resolved through dialog and, if necessary, with the assistance of a third author.

**Fig. 1:**
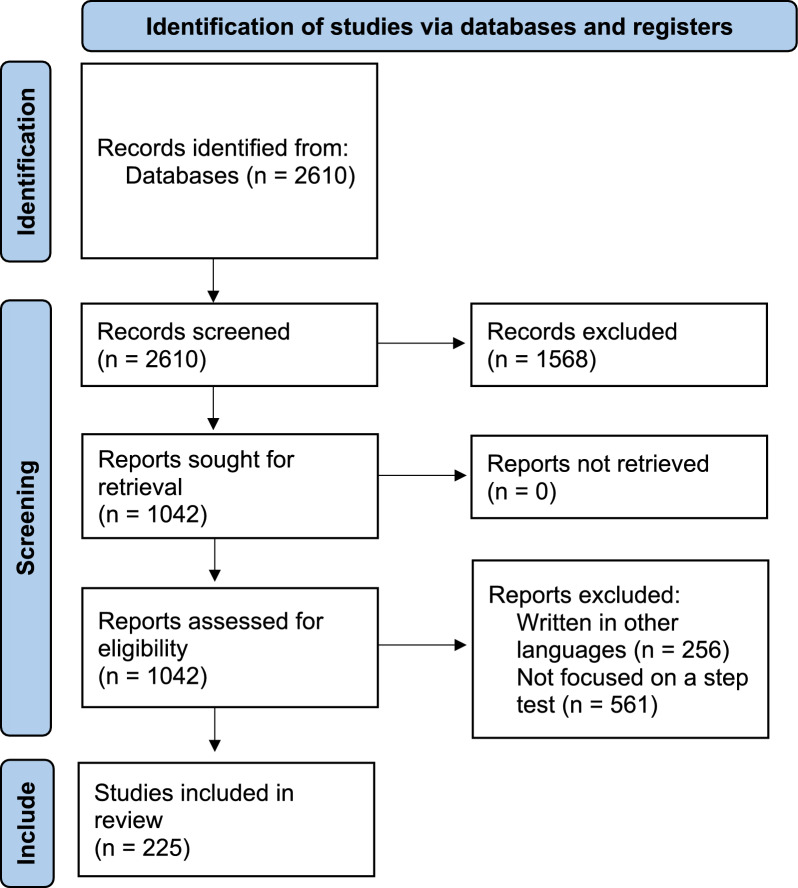
Flowchart of the review

The Web of Science search yielded 2610 records. 1568 studies were excluded after evaluating the title and abstract. The remaining 1042 studies were then assessed by reading the relevant sections. 817 studies that did not meet the inclusion criteria were excluded. Finally, a total of 225 articles met the defined criteria and were included in the review. Figure 1 shows the identification, screening, and inclusion of the articles from the database for the review. Additional information regarding the included studies can be found in the supplementary file.

### Analytical Methods and Tools

Bibliometric data extraction and analysis were performed using specialized bibliometric software. The VOSviewer, a Java-based measurement tool developed by Van Eck and Waltman, specializes in co-occurrence network clustering and density analysis [23]. By visualizing co-occurrence and co-sponsorship networks, different clusters are visually represented by different colors, while lines between nodes indicate collaborative relationships. In particular, in the average publication year graph, colors correspond to different years, which facilitates temporal analysis.

Therefore, VOSviewer was used to extract relevant bibliographic information for each included publication including title, authors, year of publication, journal, keywords, number of citations, and abstract. In cases where full-text articles were available, additional data such as methodology and results were extracted for detailed analysis. Visualization techniques, including co-authorship networks, and keyword clustering, were used to provide a comprehensive overview of the step test literature and its evolution.

### Analysis of Publication Outputs

**Fig. 2:**
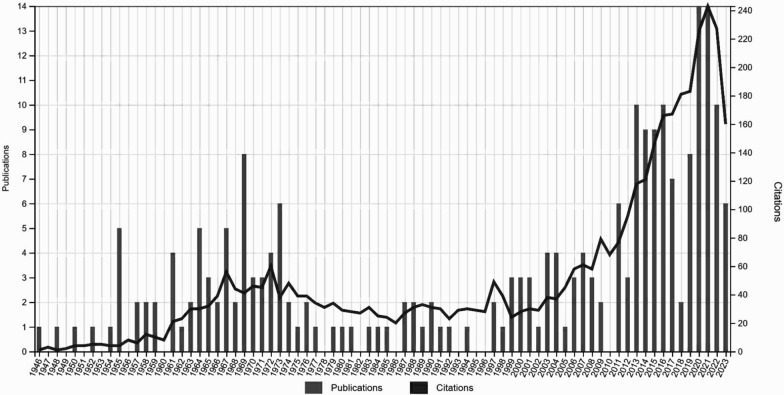
Annual number of step test publications and citations per year

Between 1946 (year of the first publication involving a step test) and 2023, the WoS database contained a total of 248 papers. Figure 2 illustrates the publication output of step test research from 1946 to 2023. The annual publications showed a tendency to increase over the years from one publication in 1946 to fourteen in 2022. Notably, the largest number of articles was published in 2020 and 2021, both years with 14 papers per year. Also, 2021 stands out for having the most extensive publications and the greatest number of citations (14 publications and 243 citations).

Publications related to the step test show a segmented evolution into four distinct phases according to publication numbers: the initial phase (1946–1969), the second phase (1969–2011), the third phase (2011–2021), and the fourth phase (2021–2023). The step test gained significant interest in 2011 due to important scientific advances. However, prior to that year, there had been an overall decline in the number of publications. Subsequently, in 2013, the number of publications reached 10 for the first time. From then on, the volume of literature increased steadily, following a consistent upward trajectory, and breaking the 14-mark for the first time in 2020. However, the last two years have seen a decline in the number of publications.

**Fig. 3:**
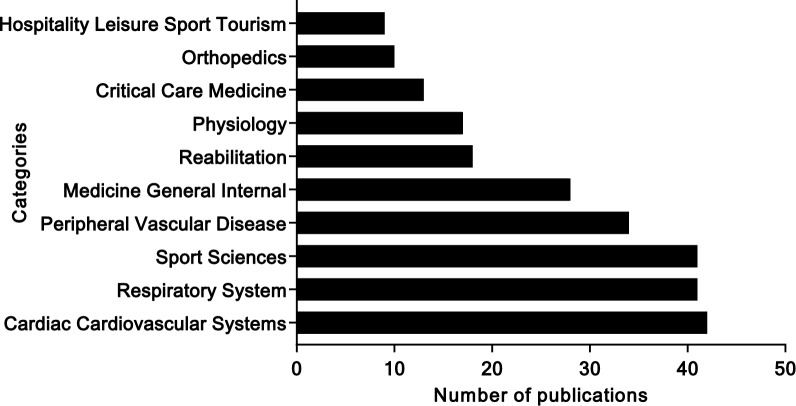
Top 10 categories for publications on the step test

Figure 3 shows the analysis of the categories. The top-ranked fields are Cardiovascular (n = 42 publications), Respiratory System (n = 41 publications), Sport Sciences (n = 41 publications), Peripheral Vascular Disease (n = 34 publications), General Internal Medicine (n = 29 publications), Rehabilitation (n = 18 publications), Physiology (n = 17 publications), Critical Care Medicine (n = 13 publications), Orthopedics (n = 10 publications), and Hospitality Leisure Sport Tourism (n = 9 publications).

**Fig. 4:**
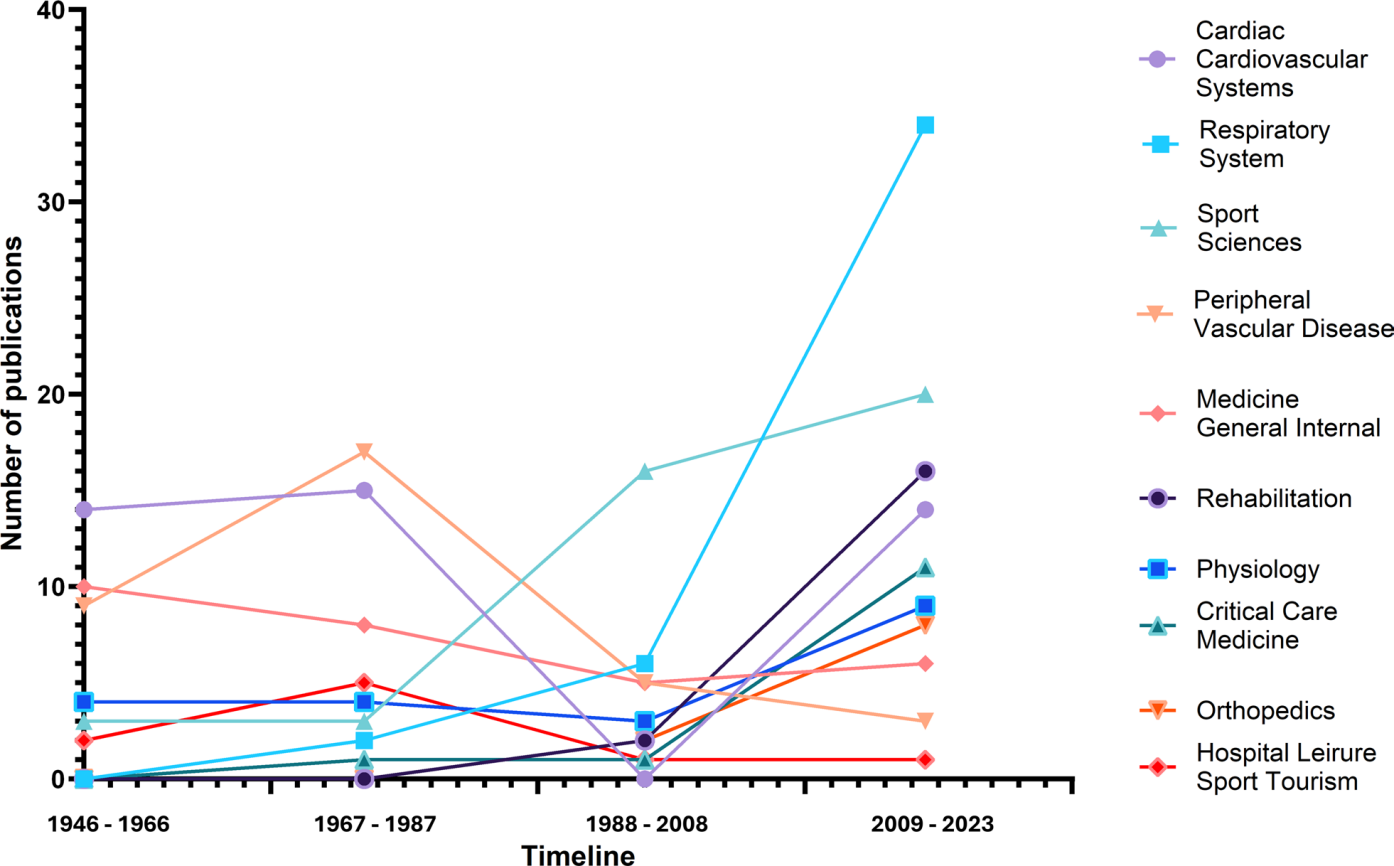
Major changes in the top 10 categories for publications on the step test over the years

The frequency of the top ten categories in terms of publications and citations between 1946 and 2023, with intervals of 20 years, in relation to the step test indexed in the Web of Science core collection is shown in Fig. 4.

### Analysis of Countries and Institutions

**Fig. 5:**
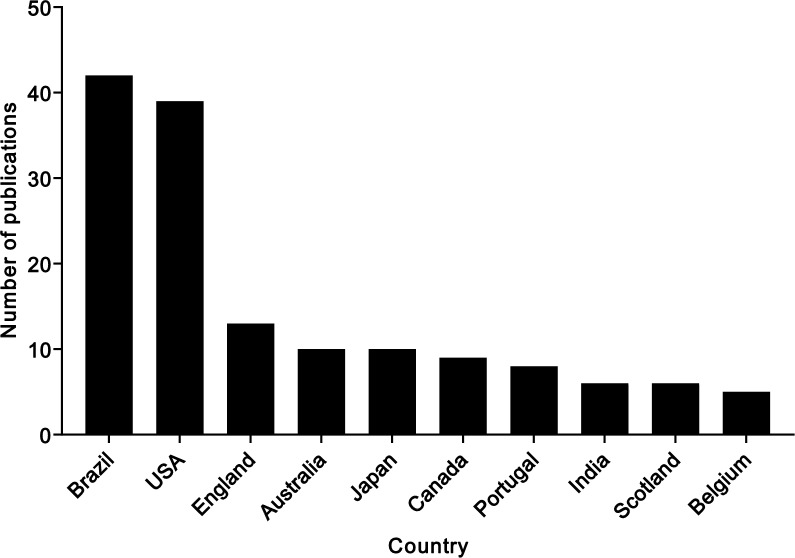
Top 10 productive countries for publications on the step test

According to the corresponding author’s country, a total of 31 countries and regions contributed to the cardiovascular assessment using the step test. In the period from 1946 to 1973, only 3 countries (United States of America, Japan, and Canada) contributed to the step test results, whereas in the period from 2013 to 2023, researchers from 27 countries were involved in such research. The top ten countries and regions were: Brazil contributed the most (n = 42 publications), accounting for 19% of the total publications, followed by the United States of America (USA, n = 39), England (n = 13), Australia (n = 10), Japan (n = 10), Canada (n = 9), Portugal (n = 8), India (n = 6), Scotland (n = 6), and Belgium (n = 5) (Fig. 5).

**Fig. 6:**
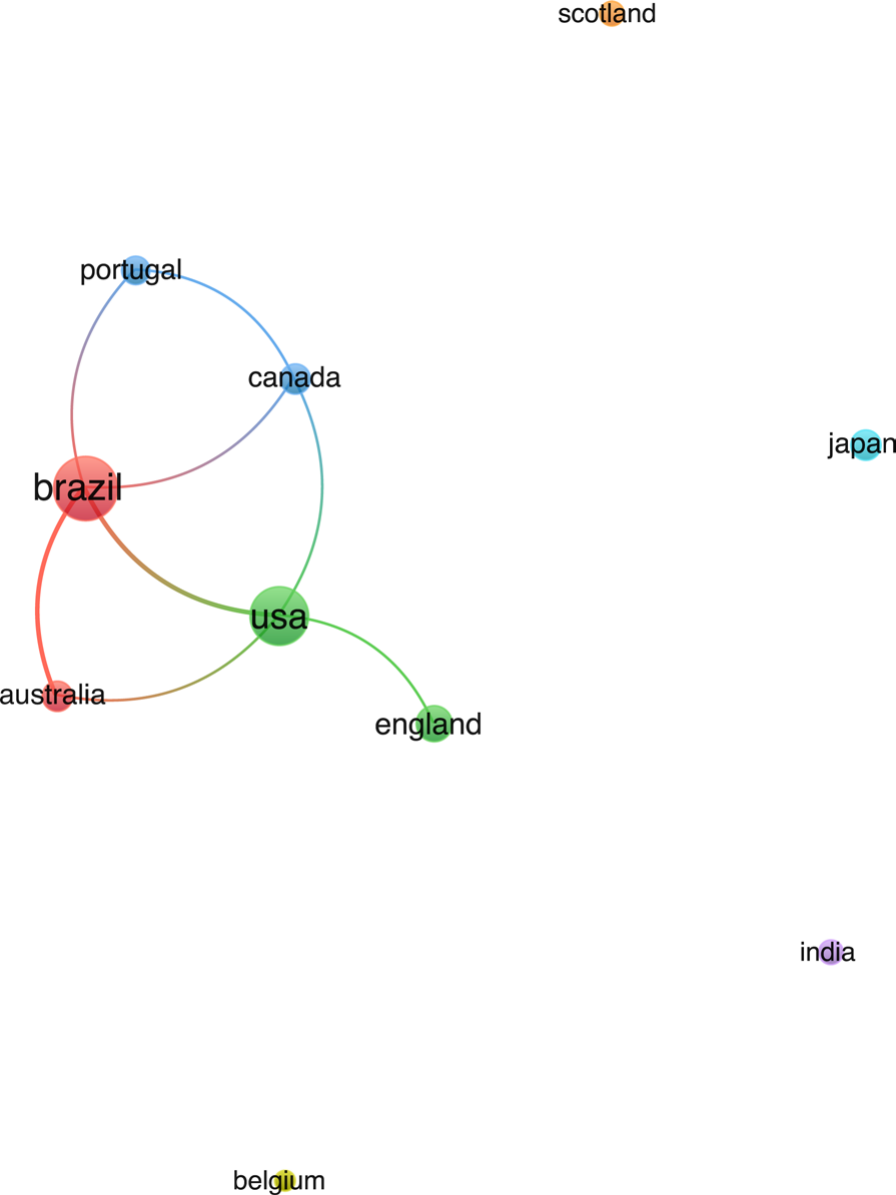
The cooperation network visualization map of step test research publishing institutions based on VOSviewer. Distinct clusters are visually represented by different colours, while lines between nodes denote collaborative relationships

**Fig. 7:**
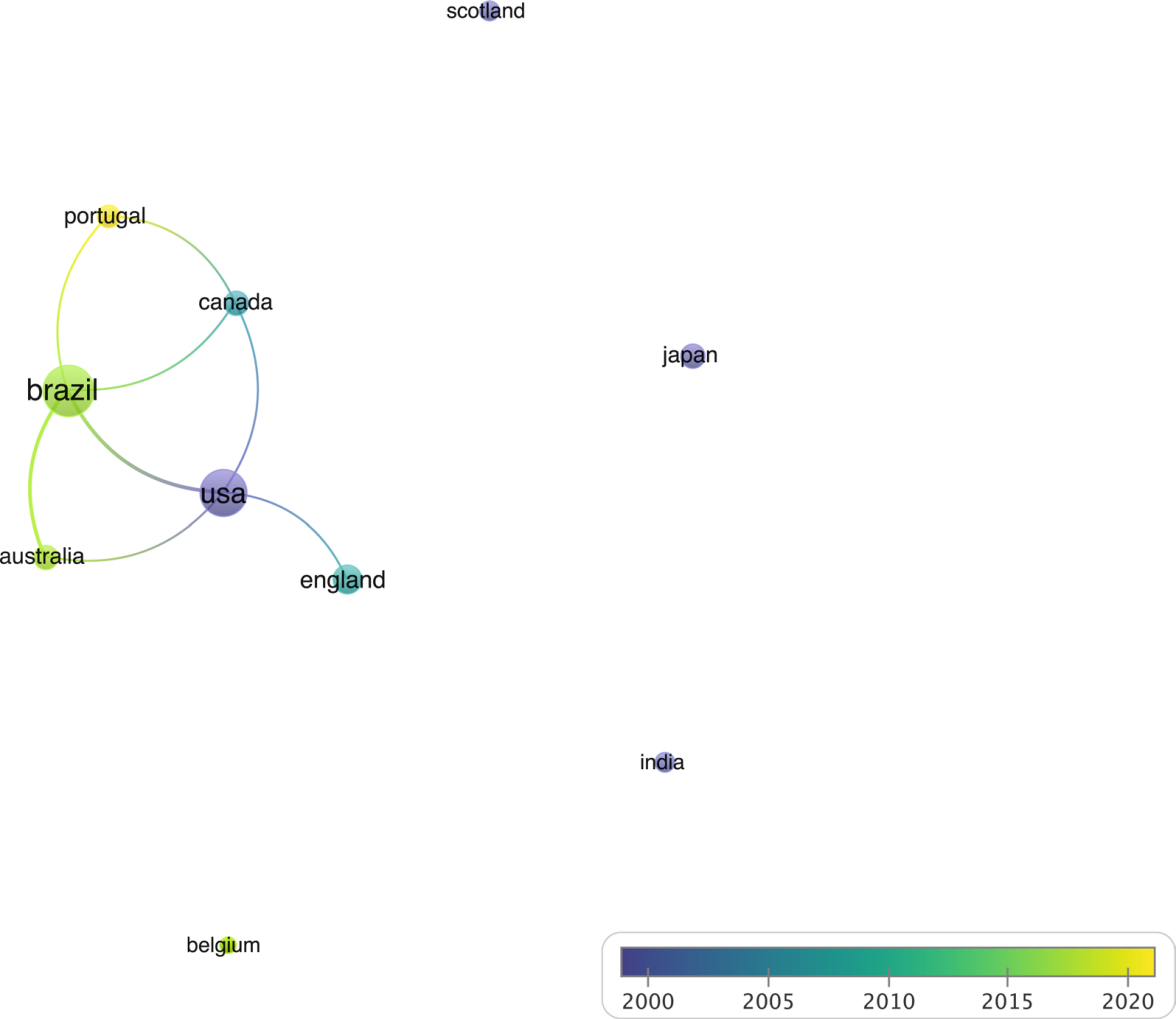
The cooperation network visualization map of step test research publishing institutions based on VOSviewer with the timeline. In the average publication year graph, colours correspond to different years, aiding in temporal analysis

Figures 6 and 7 show the co-authorship analysis by country and provide dynamic visualizations of collaborative networks in step test research over time. Specifically, Fig. 6 presents the co-authorship network with clustering colors, where different clusters are highlighted. This visualization allows for the identification of distinct groups of collaborating countries and offers insights into the primary clusters within the research community. The research landscape in this domain was mainly a divided network of individual clusters, with the presence of three clusters constituted by six members (Portugal, Canada, Brazil, USA, England, and Australia) being particularly noteworthy.

Figure 7 depicts the same co-authorship network but employs a timeline overlay, with nodes colored according to specific years. By superimposing the timeline on the co-authorship network, this figure illustrates the evolution of collaborative patterns between countries over different time periods. The varying thickness of the lines and the positioning of the nodes indicate the strength and changes in international collaborations over time. Visual inspection reveals that none of these countries are actively collaborating (Fig. 7).

A total of 1849 institutions participated in research on the step test as a tool for cardiovascular assessment between 1946 and 2023. The top ten institutions contributing to step test research are shown in Table 1.

**Table 1:** Top 10 institutions contributing to step test research

Rank	Institution	Articles	Country
1	University of Alabama System	16	USA
2	Universidade Nove de Julho	14	Brazil
3	Universidade Federal de São Carlos	11	Brazil
4	Universidade do Estado do Rio de Janeiro	6	Brazil
5	Universidade Federal de São Paulo	6	Brazil
6	Bangor University	5	UK
7	Monash University	5	Australia
8	University of Dundee	5	UK
9	City University of New York	4	USA
10	Icahn School of Medicine at Mount Sinai	4	USA

USA: United States of America; UK: United Kingdom

### Analysis of Journals

A total of 111 journals were involved in publishing cardiovascular assessments using the step test. The top ten journals are: Respiratory Care (6 documents and 102 citations), European Respiratory Journal (11 documents and 98 citations), Pediatric Pulmonology (5 documents and 69 citations), Medicine and Science in Sports and Exercise (10 documents and 188 citations), Research Quarterly for Exercise and Sport (7 documents and 46 citations), Circulation (20 documents and 56 citations), Journal of the American Medical Association (6 documents and 122 citations), European Journal of Applied Physiology (5 documents and 54 citations), American Journal of respiratory and Critical Care Medicine (6 documents and 6 citations), and Japanese Circulation Journal (English edition) (5 documents and 0 citations).

### Analysis of Authors

**Fig. 8:**
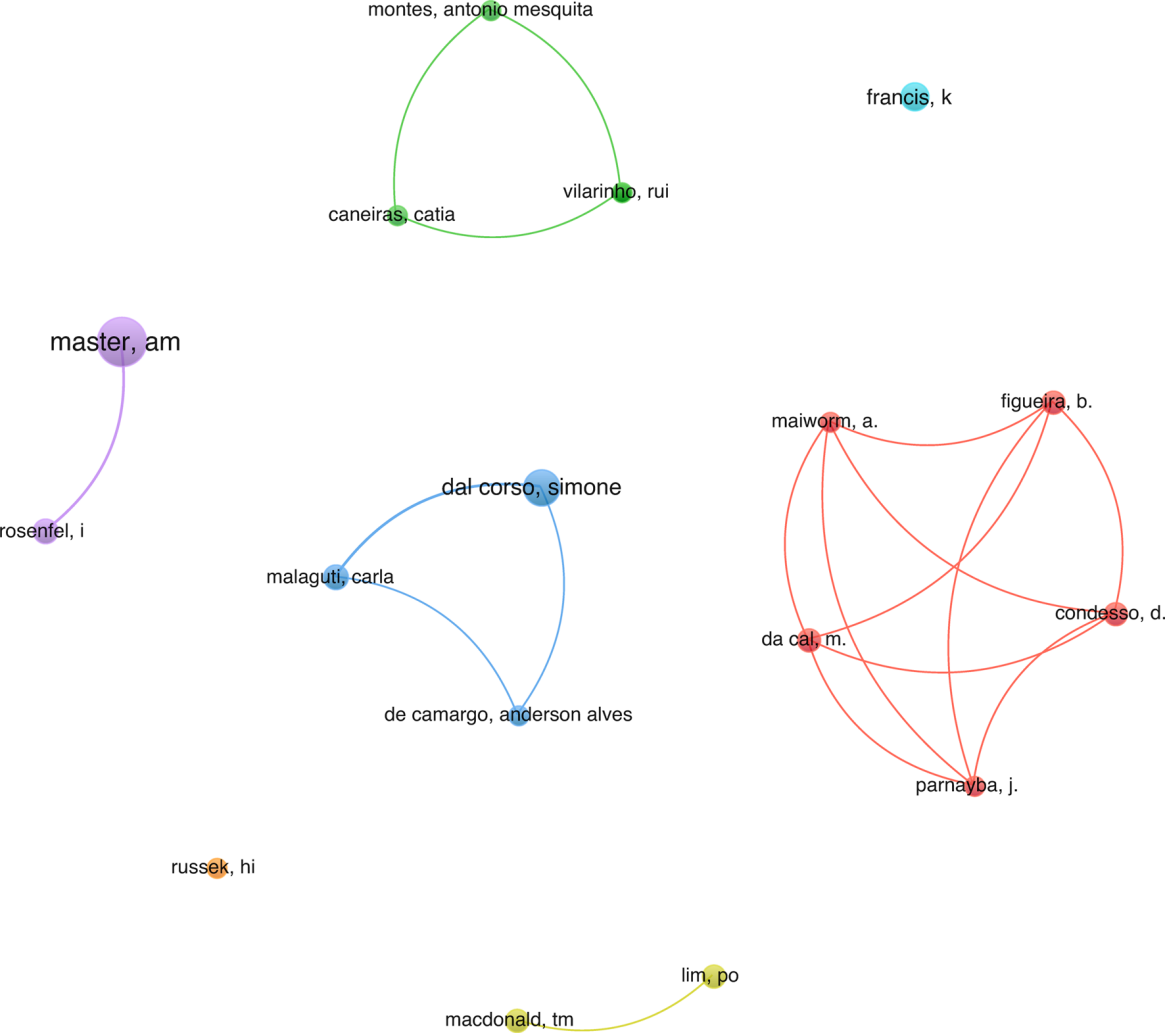
The cooperation network visualization map of step test research publishing authors based on VOSviewer

Since 1946, 761 researchers have contributed to the advancement of research in this specific field. The use of visualization maps can provide valuable insight into potential collaborators, helping researchers establish productive partnerships. Using a threshold of four documents per author, Fig. 8 allows the visualization of seven distinct clusters. As shown in this figure, the research landscape in this domain has not yet coalesced into a tightly knit network; instead, it shows a diverse range of authors with some lower output authors. Noteworthy is the presence of a 12-member team actively participating in small-scale collaborations, as highlighted in Fig. 7.

### Analysis of References

Since 1946, publications in this area have been cited 4,037 times, with an average of 51.76 ± 1.42 citations per year. The five most cited references are presented in Table 2, with the highest number of references being to the authors Åstrand and Ryhming for their article entitled “A Nomogram for Calculation of Aerobic Capacity (Physical Fitness) From Pulse Rate During Submaximal Work” [25].

**Table 2:** The top 5 cited references

Rank	Title	Authors (year)	Citations
1	A Nomogram For Calculation Of Aerobic Capacity (Physical Fitness) From Pulse Rate During Submaximal Work [25]	Åstrand and Ryhming (1954)	1373
2	Reliability And Interrelationships Between Maximal Oxygen Intake, Physical Work Capacity And Step-Test Scores In College Women [6]	Mcardle et al. (1972)	183
3	Can Primary Care Doctors Prescribe Exercise to Improve Fitness? The Step Test Exercise Prescription (Step) Project [26]	Petrella et al. (2003)	140
4	Reliability And Validity Of Measures Taken During The Chester Step Test To Predict Aerobic Power And To Prescribe Aerobic Exercise [27]	Buckley et al. (2004)	109
5	A Simple, Valid Step Test For Estimating Maximal Oxygen-Uptake In Epidemiologic Studies [28]	Siconolfi (1985)	98

### Analysis of Keywords

**Fig. 9:**
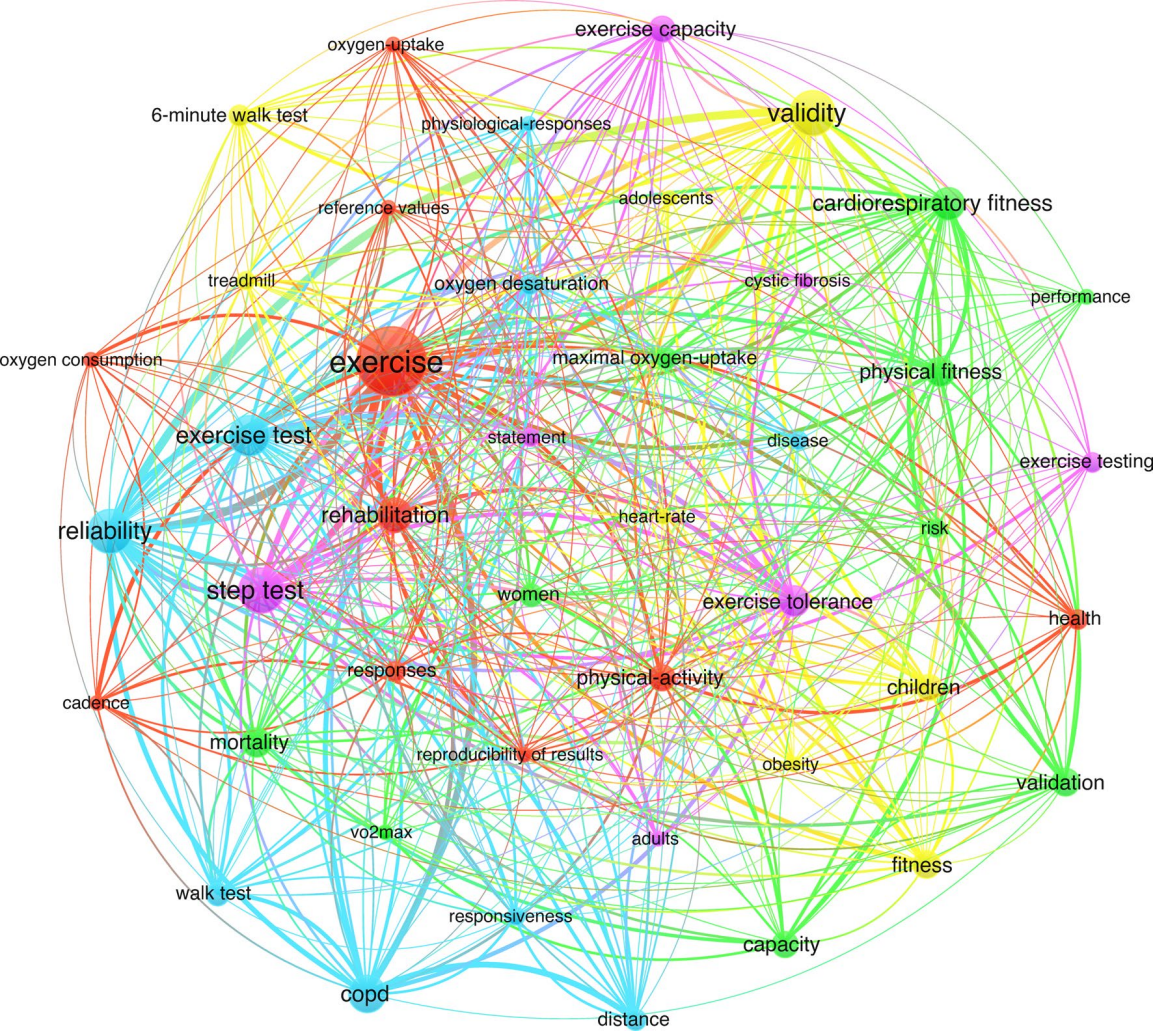
Step test research publication keywords clustering map based on VOSviewer. Distinct clusters are visually represented by different colours, while lines between nodes denote collaborative relationships

**Fig. 10:**
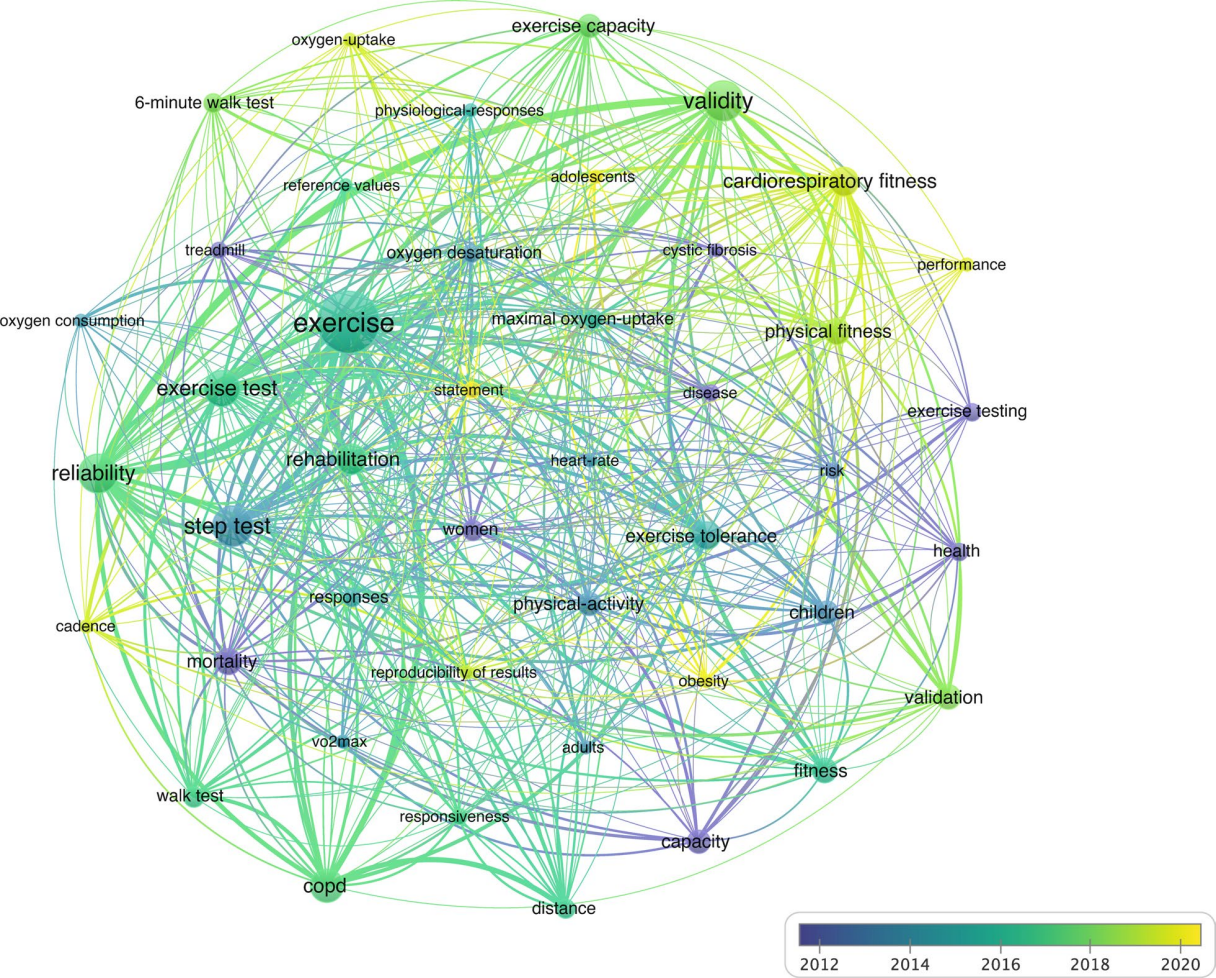
Step test research publication keywords clustering map with the timeline based on VOSviewer. Distinct clusters are visually represented by different colours, while lines between nodes denote collaborative relationships. In the average publication year graph, colours correspond to different years, aiding in temporal analysis

Co-occurrence clustering of keywords can help identify emerging trends and patterns in the development of a topic, as well as 'hot' areas in the field of study. It can reveal the research frontier of the field and the internal organization of an academic field. Figures 9 and 10 illustrate keyword co-occurrence analysis; by applying a threshold of at least five occurrences per keyword, it is possible to visualize distinct clusters.

Specifically, Fig. 9 employs clustering colors to visualize distinct clusters based on keyword co-occurrence. Each cluster represents a different research topic, facilitating the identification of various areas of focus within the field. Larger dots correspond to keywords with a greater number of occurrences, indicating hotspots in the research domain. The connections between nodes represent the strength of associations, with a higher number of lines signifying a greater co-occurrence of keywords in the same articles. Thus, the clusters emerged with the following keywords: cluster 1 (cadence, exercise, exercise testing, health, and oxygen uptake); cluster 2 (capacity, cardiorespiratory fitness, maximal oxygen uptake, and mortality); cluster 3 (chronic obstructive pulmonary disease, exercise capacity, and physiological response); and cluster 4 (adolescents, children, fitness, and obesity), cluster 5 (as adults, cystic fibrosis, exercise testing, and exercise tolerance). Note that these listed keywords are representative and do not encompass the full range of terms present within each cluster.

Figure 10 depicts the same keyword co-occurrence network, incorporating a timeline. Nodes are colored according to the time of appearance, ranging from blue to yellow. This temporal overlay highlights the evolution of research topics over time, illustrating how trends and focus areas have developed. The varying thickness of the edges and the sizes of the nodes indicate the intensity and shifts in keyword co-occurrence, reflecting changes in research trends over different periods.

### Comprehensive Analysis

Bibliometric analysis of the use of the step test to assess cardiorespiratory fitness in recent years shows a gradual increase in the number of publications until 2021. Although a decrease was observed in the last 2 years, the analysis of research categories underlines the multidisciplinary nature of the step test studies. Primarily anchored in fields such as cardiovascular, respiratory, sports sciences, and medicine, the step test has gained attention across disciplines. As these disciplines evolve, the exploration of the step test applications in various occupational and clinical settings has gained prominence, demonstrating its relevance in different contexts.

The global distribution of research reveals collaborative efforts from 31 countries and regions, underscoring the worldwide importance of the step test. However, there is a notable concentration of research activity with Brazil and the United States emerging as “key” leaders. Encouragingly, the diversity of contributing nations has increased over time, suggesting a globalizing interest in step test research.

With 111 journals contributing to the step test literature, the analysis demonstrates the dispersed nature of research dissemination findings across the academic landscape. Notable among these are journals such as Respiratory Care, European Respiratory Care Journal, and Medicine and Science in Sports and Exercise, which demonstrate the interdisciplinary nature of the step test studies. Researchers seeking valuable references and insights into step test research may find these journals particularly influential.

The extensive list of 761 researchers contributing to step test research represents a collaborative and diverse community. The visualization of author clusters reveals both established collaborations and emerging networks, highlighting the dynamic nature of research partnerships. Notably, the top 10 institutions contributing to step test research include the University of Alabama System, Universidade Nove de Julho, and Monash University, among others. These institutions represent a mix of academic and research centers, demonstrating the diverse settings in which step test studies are conducted.

Exploration of the research main points within step test studies using co-occurrence keyword analysis has revealed distinctive clusters that indicate emerging trends that significantly guide the ongoing research in cardiovascular assessment. This comprehensive analysis serves as a critical tool for identifying emerging research frontiers and strategically guiding future investigations, thereby promoting a nuanced understanding of the critical issues influencing step test research.

Through bibliometric analysis of the step test literature, five clusters have emerged, each delineated as suggested: (1) Health and Exercise Guidelines, (2) Cardiovascular Fitness and Mortality Risk, (3) Chronic Obstructive Pulmonary Disease (COPD) and Respiratory Response, (4) Pediatric Exercise and Fitness, and (5) Adult Populations and Specific Conditions.

In the first cluster, related to health and exercise guidelines, the convergence of keywords such as cadence, exercise, exercise testing, health, and oxygen uptake indicates a significant emphasis on standardizing protocols, establishing reference values, and formulating guidelines for conducting step tests [29, 30]. The inclusion of terms such as rehabilitation and reproducibility of results underscores ongoing efforts to refine and improve the reliability of step test results. Protocols such as the StepTest4all [8] involved participants in a continuous progressive test on a stable step, alternating between ascending and descending. The step height was individually determined based on factors such as gender, age, physical fitness, height, body mass index, and smoking status. The formula, derived from various tests, takes into account factors that influence cardiovascular fitness. Protocols such as the StepTest4all proposed by Bragada et al. [8] contribute to a broader applicability in health and exercise contexts, providing a reliable framework to assess cardiovascular capacity and overall health.

In the second cluster, cardiovascular fitness and mortality risk, keywords such as capacity, cardiorespiratory fitness, maximal oxygen uptake, and mortality form a distinct focus. This indicates a strong emphasis on elucidating the cardiovascular implications of step test results. Research within this cluster is likely to explore the intricate relationship between step test outcomes and broader health considerations, particularly mortality risk [14]. So et al. [31] determined the frequency of cardiovascular disease risk occurred among Japanese workers along with how the Japan step test evaluated cardiovascular fitness related to that risk. The Japan step test evaluated cardiovascular fitness was found to be inversely correlated with the prevalence of cardiovascular disease risk. For the purpose of identifying workers who may develop cardiovascular disease, the Japan step test may be useful [31]. Additionally, the presence of terms such as validation and VO_2max_ underscores a careful analysis of the validity of step test measures in assessing cardiovascular fitness.

In the third cluster, chronic obstructive pulmonary disease and respiratory response, keywords such as chronic obstructive pulmonary disease, exercise capacity, and physiological response indicate a special focus on respiratory aspects and the application of the step test in COPD populations [32–36]. The Chester step test was performed using a 20 cm tall, handle-less, single-step device. This test consists of five steps, each lasting 2 min, for a total test duration of 10 min. The step cadence, initially set at 15 steps/min, increases by 5 steps/min every 2 min through the stages: 15 steps/min in stage 1, 20 steps/min in stage 2, 25 steps/min in stage 3, 30 steps/min in stage 4, and 35 steps/min in stage 5. Notably, the Chester step test has been found to be highly reproducible in patients with COPD, as demonstrated by de Camargo et al. [37], further emphasizing the relevance and efficacy of the step test in this specific context. The exploration of oxygen desaturation, reliability, and responsiveness within this cluster reflects a focused effort to understand and improve the utility of the step test in assessing respiratory health.

In the fourth cluster, pediatric exercise and fitness, the inclusion of keywords such as adolescents, children, fitness, and obesity underscores a particular interest in the application of the step test in younger populations [38–41]. The emphasis on validity and heart rate within this context suggests ongoing efforts to refine and validate step test protocols specifically adapted for assessing fitness and health in children and adolescents. For example, the Chester step test has been shown to be versatile, as illustrated by Maggio et al. [38], who demonstrated its ability to assess cardiorespiratory fitness in children in a clinical setting, highlighting its applicability to diverse pediatric populations.

Finally, in the fifth cluster, adult populations and specific conditions, keywords such as adults, cystic fibrosis, exercise testing, and exercise tolerance indicate research specifically directed at unique health conditions within the adult population. The inclusion of cystic fibrosis signals a specific focus on the application of the step test to populations facing unique health challenges [20, 42, 43]. This cluster likely navigates the adaptability and efficacy of the step test in diverse adult populations. In particular, the work of Holland et al. [42] demonstrates that desaturation during the 3-min step test is associated with long-term lung deterioration and increased hospital days in adults with cystic fibrosis. Holland et al. [42] suggested that the 3-min step test may be a useful screening tool for patients with moderate to severe cystic fibrosis lung disease, highlighting its potential as a valuable measure for identifying individuals who require increased intervention and monitoring.

The clusters identified in this bibliometric analysis of the step test literature provide a guide for future research. The health and exercise guidelines cluster suggests methods for refining protocols and ensuring reproducibility. The cardiovascular fitness and mortality risk cluster provides opportunities to explore the relationship between step test results and broader health implications. The chronic obstructive pulmonary disease and respiratory response cluster may focus on optimizing the step test for COPD populations. The pediatric exercise and fitness cluster highlights the need for research on step test protocols for children, and the adult populations and specific conditions cluster suggests exploring the adaptability of the step test in diverse adult populations. These clusters guide future investigations to refine protocols, validate measures, and expand the use of the step test in different health contexts.

### Practical Implications

This bibliometric review of step test literature provides not only quantitative data on publication trends, but also valuable insights into the practical implications of incorporating the step test into various settings. These practical implications, derived from the collective findings of numerous studies, can have a significant impact on clinical decision-making, exercise prescription, and health assessment. The numerous publications related to the cardiovascular system and fitness underscore the practical application of the step test in cardiovascular risk stratification [14]. By utilizing step test results, healthcare professionals can gain a nuanced understanding of an individual's cardiovascular fitness, which can aid in the identification of those at higher risk for cardiovascular events. This, in turn, facilitates personalized interventions and lifestyle modifications to reduce cardiovascular risk. The emphasis on the use of the step test in specific populations, including adolescents [38] and individuals with disease [37], underscores the importance of these protocols as non-invasive tools for assessing exercise capacity and overall health. The integration of the step test into routine assessments is proving to be a valuable tool for monitoring respiratory function and optimizing treatment approaches for individuals with respiratory disease. Similarly, incorporating the step test into pediatric health assessments will be critical in guiding interventions that promote lifelong health and well-being, especially in healthcare settings such as hospitals. The focus on COPD and exercise capacity highlights the practical utility of the step test in monitoring respiratory health. Particularly relevant in populations with respiratory disease, step tests provide a non-invasive means of assessing exercise capacity [37]. Integrating the step test into routine assessments can facilitate the tracking of respiratory function over time and the optimization of treatment strategies for individuals with respiratory disease. In the context of adolescents, children, and fitness, the cluster highlights the practical implications of step tests in pediatric fitness assessments. Recognizing the unique considerations for younger populations, the step test provides a feasible and informative tool for assessing fitness levels in children and adolescents [38]. Incorporating the step test into pediatric health assessments can guide interventions aimed at promoting lifelong health and wellness.

In addition, in the field of sport sciences, the step test is a valuable tool to optimize exercise prescription. The knowledge gained from these studies contributes to the development of personalized exercise programs that take into account individual fitness levels and goals. The practical implication is a more targeted and effective approach to exercise interventions, whether in rehabilitation settings or athletic training programs [44].

The concentration of publications in rehabilitation indicates the practical importance of the step test in informed exercise interventions. Whether in cardiac rehabilitation or broader rehabilitation programs, the step test provides valuable data on an individual’s exercise capacity and response [45]. This information guides the design of individualized rehabilitation protocols and optimizes the effectiveness of therapeutic interventions.

### Limitations and Further Research

Despite the aforementioned advantages of the present research, the bibliometric methodology has some inherent limitations. The decision to consider only English-language publications introduces a language bias, potentially excluding relevant research published in other languages and limiting the overall scope of the investigation. Additionally, the search strategy that relied primarily on the terms "step-test" or "step test" may have inadvertently omitted relevant articles that did not explicitly include these specific terms in the title or abstract, potentially missing valuable contributions to the field. Future research should consider broader language inclusivity and employ a more comprehensive search strategy to improve the inclusivity of the bibliometric analysis.

## Conclusions

In conclusion, this bibliometric analysis of the step test literature reveals a dynamic landscape characterized by a gradual increase in publications until 2021, signaling a sustained interest in this cardiovascular assessment tool. Despite a recent decline in the last two years, the multidisciplinary nature of step test studies is evident, anchored in fields such as cardiovascular, respiratory, sports sciences, and clinical medicine. This breadth of research underscores the relevance of the step test in diverse areas, reflecting its adaptability and ease of application in occupational and clinical settings.

### Supplementary Information

Additional file 1.

## Data Availability

Data are available on request from the contact author.

## Abbreviations

METs: Metabolic equivalents
VO_2max_: Maximal oxygen consumption
HRR: Heart Rate Recovery
WoS: Web of Science
UNIFESP: Universidade Federal de São Paulo
COPD: Chronic Obstructive Pulmonary Disease
