# Supplementary material for: Bibliometric Review of the Step Test: A Comprehensive Analysis of Research Trends and Development
Source: Sports Med Open. 2024 Aug 28;10:91. doi: 10.1186/s40798-024-00764-y (PMC11358565; doi:10.1186/s40798-024-00764-y)
Supplement: Supplementary file 1 — Additional file 1. [file 40798_2024_764_MOESM1_ESM.docx]

Supplementary file

Table. Included studies characteristics.

| Study | Aim | Sample characteristics | | | Step test protocol |
| --- | --- | --- | --- | --- | --- |
|  |  | N | Age (M ± SD) | Sex |  |
| [1] | To examine a modification on a standard step test allowing for the use of a pre-existing in-home step of variable height for cardiorespiratory fitness testing | 37 | 24 ± 5 | Male = 14 Female = 23 | NA |
| [2] | To match the VO_2_ exercise intensity to an established step test (YMCA step test) by altering the step frequency to compensate for the height of available in-home objects used as steps | 40 | 24 ± 5 | Male = 16 Female = 24 | YMCA step test |
| [3] | To define standardized results of the master two step test in patients with coronary artery disease | 24 | NA | NA | Master two step test |
| [4] | To compare the value of radio electrocardiography during double master 2-step test compared with standard post-exercise electrocardiogram | NA | NA | NA | Master two step test |
| [5] | To derive an equation to predict 6-minute walk test from two-minute step test and to observe the agreement between observed and estimated 6-minute walk test distances | 51 | 72.9 ± 4.6 | Male = 24 Female = 27 | Two-minute step test |
| [6] | To test this model in a broader age range and in individuals with Alzheimer’s disease | 102 | NA | Male = 48 Female = 54 | The step test exercise prescription |
| [7] | To assess the concurrent validity of the number of steps on the 6-minute step test in physical capacity of patients with COPD using the 6-minute walk test as the gold-standard test | 32 | 69 ± 10 | NA | 6-minute step test |
| [8] | To examine the effect of altitude on the fitness score of the three-min step test, and the role of ambient temperature in this effect | Study 1 = 11  Study 2 = 23 | Study 1: 18.1 ± 1.1  Study 2: 20.4 ± 0.4 | Study 1: Male = 7; Female = 4  Study 2: Male = 19; Female = 4 | Three-minute step test |
| [9] | To systematically review literature on the validity and reliability of submaximal step-test protocols to estimate VO_2max_ in healthy adults | NA | NA | Both | Chester step test, a personalised step test, the step tool step test, the queen’s college step test, the skubic and hodgkins step test, a height-adjusted, rate-specific, single-state step test, the astrand–ryhming step test, and a modified YMCA 3-min step test |
| [10] | To assess the suitability of the queen’s college step test to predict maximum oxygen uptake in Indian men | 30 | 22.6 ± 0.2 | NA | Quenns colege step test |
| [11] | To validate the applicability of queen’s college step test for indirectly estimating the maximum oxygen uptake in female sedentary university students | 40 | 21.9 ± 3.2 | NA | Quen’s collgee step test |
| [12] | To determine whether maximal oxygen uptake could be predicted accurately and reliably from a two-step, perceptually regulated exercise test in healthy adults | 16 | 31.7 ± 11.3 | Male = 13 Female = 3 | Perceptually regulated exercise test |
| [13] | To examine the validity and reliability of a submaximal step test for use in rheumatoid arthritis patients | 30 | 53 ± 10 | Male = 6 Female = 24 | The siconolfi step test |
| [14] | To validate, in this population, the siconolfi step test developed to predict VO_2max_ in healthy individuals. | 30 | 48 ± 14 | Male = 5 Female = 25 | The siconolfi step test |
| [15] | To determine the 6-minute step test's reliability and validity and to establish reference performance values of this test | 91 | 39 ± 17 | Male = 42 Female = 49 | 6-minute step test |
| [16] | To assess the validity and reliability of the 6-min step test in individuals with coronary artery disease | 35 | 65.8 ± 9.6 | Male = 23 Female = 12 | 6-minute step test |
| [17] | To develop a new incremental and externally paced step test, and assess its validity in people with COPD | 50 | 70.8 ± 7.5 | Male = 28 Female = 22 | Incremental step test |
| [18] | To test the validity of the prediction equation for vo2max from the step tool for use with adults older than 65 years | 40 | 43 ± 14 | Male = 20 Female = 20 | Step tool |
| [19] | To validate the kasch 3-minute step test | NA | NA | NA | Kasch 3-minute step test |
| [20] | To test the validity of 3 different submaximal tests, 3-min step test with 30-cm step box height, and 6-min walk test in estimating maximal oxygen consumption in young and healthy individual | 73 | 30.8 ± 9.3 | Male = 37 Female = 36 | 3-minute step test |
| [21] | To validate a specific step test (steptest4all) as an adequate procedure to estimate cardiovascular capacity in young adults | 56 | Male = 22.05 ± 3.14  Female = 21.05 ± 2.39 | Male = 37 Female = 19 | Steptest4all |
| [22] | To validate the siconolfi step test to predict VO_2max_ | NA | NA | NA | The siconolfi step test |
| [23] | To develop a valid equation to estimate VO_2max_ from a fixed-rate step test in a larger sample of healthy adults | 112 | 45 ± 13 | Male = 59 Female = 53 | Adapted version of the harvard step test |
| [24] | To determine the validity of a brief, submaximal, variable height, step test used to predict VO_2max_ | 113 | 24.3 ± 5.93 | Male = 67 Female = 46 | 4 min step test |
| [25] | To analyse the individual physiological response during the YMCA step test using continuous heart rate monitoring and gas exchange measurement | 97 | 55.7 ± 14.2 | Male = 55 Female = 56 | YMCA step test |
| [26] | To validate the 3-minute Hight-adjusted step test | NA | NA | NA | 3-minute hight-adjusted step test |
| [27] | To establish the validity of the 6-minute step test as a tool to measure functional exercise performance in children without cardio-respiratory limitation by correlating them to the 6-minute step test | 74 | 8.0 ± 1.3 | Male = 27 Female = 33 | 6 minute step test |
| [28] | To identify an abnormal type of response to the master two step test | NA | NA | NA | Master two step test |
| [29] | To ascertain the utility of the Chester step test in measuring exercise capacity in patients with COPD | 32 | 66 ± 8.5 | NA | Chester step test |
| [30] | To contrast the in-formation obtained from a simple submaximal test (the 3-min step test) with that from a maximal cycle ergometry study | 19 | NA | Male = 8 Female = 11 | Three min step test |
| [31] | To evaluate the responsiveness of the 3-minute constant rate step test to detect the relief of exertional dyspnea after acute bronchodilation in COPD patients | 40 | 69 ± 7 | Male = 33 Female = 7 | Three min step test |
| [32] | To validate the height adjusted, rate specific single stage step test | NA | NA | NA | The height adjusted, rate specific single stage step test |
| [33] | To describe the use of step tests for the assessment of exercise capacity in healthy subjects and in patients diagnosed with chronic lung disease | NA | NA | NA | The master two-step test, the harvard pack test, the harvard step test, the astrand-ryhming step test, gradational step tests, the queen’s college step test, the memorial hospital step test, the chester step test |
| [34] | To evaluate the validity and reliability of 3-minute step test in healthy children | 66 | 9.2 ± 1.2 | Male = 23 Female = 43 | The 3-min step test |
| [35] | To evaluate the validity and reliability of the siconolfi step test in patients with axial spondyloarthropathy | 15 | 53 ± 11 | Male = 11 Female = 4 | Siconolfi step test |
| [36] | NA | NA | NA | NA | 6-minute step test |
| [37] | To analyze the association between the 6-minute step test and peak oxygen uptake and develop an equation for estimating peak oxygen uptake based on the 6-minute step test | 171 | 60 ± 14 | Male = 121 Female = 50 | 6-minute step test |
| [38] | To evaluate the 6-minute step test as a predictor of cardiorespiratory fitness in obese women | 56 | 35 ± 7 | Female = 56 | 6-minute step test |
| [39] | To develop the 6 - minute step test standard equation for sedentary subjects | NA | NA | NA | 6-minute step test |
| [40] | To evaluate the validity and reliability for predicting cardiopulmonary fitness in men with prostate cancer | 83 | 68.2 ± 7.4 | Male = 83 | Siconolfi step test |
| [41] | To assess in patients with cardiovascular disease the psychometric properties | 70 | CG: 56.9 ± 7.6 EG: 56.1 ± 7.6 | NA | The short and fast step test |
| [42] | To determine the relationship between the step-test index and whole-body glycemic control at different age ranges. | 347 | < 30 and > 40 | NA | NA |
| [43] | To develop a standard reference equation for the step test in 6 minutes | 326 | Between 20 and 80 | NA | 6-minute step test |
| [44] | To evaluate sensitivity, specificity, and test cutoff score to predict cardiorespiratory endurance fitness levels in patients with type 2 diabetes mellitus | 100 | 68.98 ± 6.23 | Male = 39 Female = 61 | Two-minute step test |
| [45] | To investigate the differences in a graduated submaximal step test energy expenditure and estimated maximal aerobic capacity | 20 | 24.5 ± 4.1 | Male = 20 | Graduated submaximal step test |
| [46] | To determine the proper way to conduct the Harvard step test | NA | NA | NA | Harvard step test |
| [47] | To investigate the effect of exercise on the electrocardiogram in the diagnosis of coronary insufficiency | 163 | NA | Male = 88 Female= 40 | The “two-step” test |
| [48] | To examine whether the adoption of an active arm action will influence the predicted VO_2max_ | 25 | 23.0 ± 6.5 | Male = 10 Female = 15 | Chester step test |
| [49] | To evaluate the applicability of the Dundee step test for the outpatient management of hypertension | NA | NA | NA | Dundee step test |
| [50] | To determine the association of several variables and body fat percentage | 1071 | Male = 12.8 ± 0.3  Female = 12.9 ± 0.3 | Male = 405 Female = 666 | Modified Harvard step test |
| [51] | To test the validity of the Chester step test to estimate the maximal oxygen uptake in adults with hypertension | 14 | 51.9 ± 9.2 | Male = 8 Female = 6 | Chester step test |
| [52] | To determine if this test can be an alternative to assess physical capacity and exertional desaturation in different contexts where other field tests cannot be performed. | 42 | 53.8 ± 10.3 | Male = 22 Female = 20 | Chester step test |
| [53] | To evaluate the validity of the Chester step test as a predictor of disease severity in cystic fibrosis | 22 | NA | NA | Chester step test |
| [54] | To verify if the 3mst is a valid tool to measure the functional exercise capacity in healthy children | 30 | 8.6 ± 1.8 | Male = 15 Female = 15 | Three-minute step test |
| [55] | To verify the validity of the 2-step test in the diagnosis of coronary insufficiency | NA | NA | NA | 2-step test |
| [56] | To provide test-retest reliability for the Chester step test and 1 minute sit to stand tests | 42 | 51.6 ± 9.3 | Male = 20 Female = 22 | Chester step test |
| [57] | To assess the suitability Harvard step test to evaluate physical fitness in Indian females | NA | NA | NA | Harvard step test |
| [58] | To investigate the systolic and diastolic recovery following the Harvard step test | 100 | ≧18 years | NA | Harvard step test |
| [59] | To analyse the reproducibility of incremental step test in patients with COPD on different days and to test the validity of incremental step test | 12 | NA | NA | Incremental step test |
| [60] | To use submaximal testing to measure changes in cardiovascular fitness in youth who are obese following | 29 | 7 - 18 | Male = 13 Female = 16 | YMCA step-test |
| [61] | The validity and reliability of submaximal step-test protocols to estimate aerobic capacity in healthy adults in a systematic review | NA | 18 - 65 | NA | NA |
| [62] | To compare the workload of bicycle ergometer with master’s two step test | NA | NA | NA | Master’s two step test |
| [63] | To investigate the safety of the Chester step test and the modified incremental step test to assess functional capacity in patients hospitalized with acute lung diseases. | 97 | 36 - 68 | Male = 46 Female = 51 | Chester step test and modified incremental step test |
| [64] | To evaluate the reliability of the ryhming step test for prediction of aerobic capacity. | 52 | NA | NA | Ryhming step test |
| [65] | To verify the association between the stair step test with peak vo2 | 167 | 60 ± 16 | Male = 119 Female = 48 | Stair step test |
| [66] | To observate the effects of training using the Harvard step test | NA | NA | NA | Harvard step test |
| [67] | To compare 2 practical measures of functional endurance | 189 | 14 - 85 | Male = 69 Female = 120 | Two-minute step test |
| [68] | To compare heart rate and oxygen saturation in the 6-minute step test | 85 | 20 - 80 | NA | 6-minute step test |
| [69] | To compare the heart rate and oxygen saturation at the end of 1st and 6th minute | 253 | 20 - 80 | NA | 6-minute step test |
| [70] | To analyze 6-minute step test reproducibility in COPD patients | 31 | NA | NA | 6-minute step test |
| [71] | To investigate the usefulness of simple exercise echocardiography using a master’s two-step test for detecting early pulmonary arterial hypertension | 89 | 59.0 ± 5.8 | Male = 14 Female = 75 | Master’s two step test |
| [72] | To investigate chest pain occurrence with the master two step test | 153 | NA | Male = 153 | Master’s two step test |
| [73] | To analyse serum cholesterol and master 2-step test responses in coronary patients receiving | NA | NA | NA | Master’s two step test |
| [74] | To evaluate physiological changes while performing the master’s two step test | NA | NA | NA | Master’s two step test |
| [75] | To review of 2 years routine use of the step test for the indirect determination of maximal oxygen-consumption | NA | NA | NA | Master’s two step test |
| [76] | To determine the results of Harvard step-test on young athletes | NA | NA | NA | Harvard step test |
| [77] | To evaluate intra and inter-rater relative and absolute reproducibility of 6mwt and 6mst in young adults. | NA | NA | NA | 6 minute step test |
| [78] | To analyze the reproducibility of performance on the 6mst and physiological variables in subjects with COPD | 40 | 67 ± 7 | Male = 31 Female =9 | 6-minute step test |
| [79] | To assess the reliability of the Chester step test and the modified incremental step test and correlate these tests with pulmonary function, heart rate, and distance walked during the 6-min walk test | 17 | 52±17 | Male = 6 Female =11 | Chester step test |
| [80] | To evaluate 6mst reproducibility in patients with COPD | 34 | 68.5 ± 10.3 | NA | 6 minute step test |
| [81] | To reflect on and review the progress and developments in cardiology at mount sinai over a span of 50 years, with a specific focus on the 2-step test | NA | NA | NA | Two step test |
| [82] | To evaluate the reliability of a 3-min step test using two stepping cadences and assess participants' rate of perceived exertion during the test and cadence preference. | 82 | 22.1 ± 2.0 | Male = 40 Female =42 | Three-minute step test |
| [83] | To assess the reliability and validity of the Chester step test in people with interstitial lung diseases | 66 | 66 ± 13 | Male = 32 Female =34 | Chester step test |
| [84] | To determine the test-retest reliability and validity of the 6-minute step test as a potential assessment of cardiorespiratory fitness of people at risk of cardiovascular disease | 30 | 55.9 ± 9.2 | Male = 14 Female =16 | 6-minute step test |
| [85] | To test the reliability and concurrent validity of the 6mst in reduced ejection fraction patients | 27 | 60 ± 8 | Male = 11 Female =16 | 6-minute step test |
| [86] | To evaluate the reliability and validity of measures taken during the Chester step test used to predict VO_2max_ and prescribe subsequent exercise. | 13 | 22.4 ± 4.6 | Male = 7 Female = 6 | Chester step test |
| [87] | To investigate the intra-rater, inter-rater and test-retest reliability and minimal detectable change of the alternate step test when assessing people with chronic stroke | 86 | 60.40 ± 5.54 | Male = 37 Female = 49 | Alternate step test |
| [88] | To determine the reliability and interrelationships between maximal oxygen intake, physical work capacity and step-test scores | 41 | NA | Women: 41 | Three-minute step test |
| [89] | To establish reference equations for the 6-minute step test based on demographic, anthropometric, body composition, and performance variables able to predict oxygen uptake in obese individuals. | 73 | 42 ± 14 | Male = 30 Female = 43 | 6-minute step test |
| [90] | To establish a reference equation for this new incremental step test for the Portuguese adult population. | 155 | 47.8 ± 19.7 | Male = 61 Female = 94 | Incremental step test |
| [91] | To report theoretical and practical problems encountered in radio electro cardiography and to describe a simple, inexpensive technic for recording the electrocardiogram during the two-step test | 80 | NA | NA | Master’s two-step test |
| [92] | To determine the relationship between ergo-spirometry and queen’s college step test in healthy and physically active men | 52 | 17-35 | Male = 52 | Queen’s college step test |
| [93] | To clarify the relationship between the results of the Master’s double two-step test and prognosis after lung cancer surgery | 365 | 61 - 73 | Male = 193 Female = 172 | Master’s double two-step test |
| [94] | To validate a sub-maximal bench stepping cardiovascular fitness test for children | 18 | 10.28 ± 1.18 | Male = 193 Female = 172 | YMCA step test |
| [95] | To develop accurate and practical prediction models of maximal oxygen consumption using the YMCA step test | 568 | 43.5 ± 12 | Male = 294 Female = 274 | YMCA step test |
| [96] | To develop and evaluate a new progressive step test designed to predict an individual's maximal oxygen consumption | 10 | 20 - 36 | Male = 10 | Progressive step test |
| [97] | To investigate cardiorespiratory responses during the 6mst and to develop a predictive equation to estimate VO_2peak_ | 31 | 32 ± 5 | Female = 31 | 6-minute step test |
| [98] | To determine if there was a better correlation between VO_2max_ and with a shorter (15-s) stepping interval | 60 | 22.8 ± 4.8 | Male = 33 Female = 27 | The modified YMCA step test |
| [99] | To describe physiological responses during the 6-min step test in patients with COPD | 36 | 67 ± 7 | Male = 29 Female = 7 | 6-minute step test |
| [100] | To investigate the physiological responses of normotensive patients who have coronary disease when subjected to the master two-step test | NA | NA | NA | Master’s two-step test |
| [101] | To examine the physiological responses of healthy, normal males to the master two-step test | NA | NA | Male | Master’s two-step test |
| [102] | To investigate the influence of body wight on workload while performing the step test | 14 | NA | NA | Double master two-step test |
| [103] | To analyse the correlation between perceived exertion and heart rate reliability during the Chester step test | NA | NA | NA | Chester step test |
| [104] | To identity pain patterns in 2-step test | NA | NA | NA | 2-step test |
| [105] | To establish normative values and a reference equation for the number of steps climbed during the 6-minute step test in healthy adults | 468 | 41 ± 17 | Male = 198 Female = 270 | 6-minute step test |
| [106] | To assess the relationship between hypertension index and systolic blood pressure change | 153 | NA | NA | The Dundee step |
| [107] | To assess the caution in performing the two-step test | 1 | 42 | Male | Master’s two-step test |
| [108] | To assess the feasibility of the two-step test in coronary insufficient patients. | NA | NA | NA | Master’s two-step test |
| [109] | To discuss the applicability of the two-step test | NA | NA | NA | Master’s two-step test |
| [110] | To discuss the use of the two-step test as in the differential diagnosis of chest pain or pressure | 800 | NA | NA | Master’s two-step test |
| [111] | To develop a modified step test to assess cardiovascular capacity | 26 | NA | Male = 26 | Modified step test |
| [112] | To determine the effect of repetition on test duration | 83 | Male = 43.7 ± 12.3  Women = 44.4 ± 12.2 | Male = 40 Female = 43 | Modified Chester step test |
| [113] | To investigate whether a 4-min step test would be a suitable surrogate of the 6-min walk test | 186 | 65 ± 9 | Male = 113 Female = 73 | Four-minute step test |
| [114] | To analyze the heart rate response to a step test as a function of the horizontal distance between the foot to the bench, the bench height, and the lower limb segmental height | 12 | 27 ± 4 | Male = 12 | Queens college step test |
| [115] | To compare the effectiveness and diagnostic value of the master’s double 2-step test and cooper's aerobic programmed treadmill test | NA | NA | NA | Masters double 2-step test |
| [116] | To evaluate the effectiveness and diagnostic accuracy of the master two-step test in detecting coronary insufficiency | 186 | NA | NA | Master two-step test |
| [117] | To re-evaluate the effectiveness and diagnostic accuracy of the master two-step test in detecting coronary insufficiency | 186 | NA | NA | Master two-step test |
| [118] | To assess the clinical usefulness of the master two-step test | 650 | NA | NA | Master two-step test |
| [119] | To identify the practical implications of the two-step test | NA | NA | NA | Master two-step test |
| [120] | To measure cardiopulmonary and metabolic exercise indices in a 6-minute step test | 22 | 38.64 ± 6.22 | Male = 13 Female = 9 | 6-minute step test |
| [121] | To investigate the performance of the 6-minute step test in COPD patients separated into different symptomatology groups based on the cat questionnaire score | 59 | NA | NA | 6-minute step test |
| [122] | To investigate the safety, feasibility and reliability of the short and fast step test, a functional test exploring anaerobic metabolism in coronary patients during cardiac rehabilitation | 42 | 57.8 ± 10.2 | Male = 37 Female = 5 | The short and fast step test |
| [123] | To investigate and analyze the factors contributing to errors in predicting VO_2max_ | NA | NA | NA | Chester step test |
| [124] | To evaluate whether the highest heart rate achieved during the master two-step test is sufficient for accurately assessing cardiovascular capacity | NA | NA | NA | Master two-step test |
| [125] | To evaluate the YMCA step test and the veteran’s specific activity questionnaire for the estimation of cardiorespiratory fitness in the general population | 105 | 22 - 79 | Male = 53 Female = 52 | The YMCA step test |
| [126] | To evaluate the agreement between the main variables obtained in the incremental step test | 20 | 41 ± 15 | Male = 7 Female = 13 | Incremental step test |
| [127] | To investigate whether exercise systolic blood pressure is a better predictor of sustained hypertension compared to casual office blood pressure | 191 | 52 ± 13 | Male = 102 Female = 89 | The Dundee step test |
| [128] | To evaluate hemodynamics of the master two step test in hypertension and healed myocardial infarction | 16 | 31 - 52 | Male = 7 Female = 9 | Master two-step test |
| [129] | To evaluate the hemodynamic changes following master two-step test in hypertension | NA | NA | NA | Master two-step test |
| [130] | To validate a model using a 3-minute single-stage step test for predicting VO_2max_ | 17 | 19 - 33 | Female = 17 | Three-minute single-stage step test |
| [131] | To investigate the relationships between heart rate, pulmonary ventilation, and arterial lactate concentration during step tests | NA | NA | NA | NA |
| [132] | To compare males and females in their response to a standard exercise, to investigate the interrelation between heart rate before, during, and after exercise and to report percentiles for age groups | 1934 | 10 - 69 | Male = 998 Female = 936 | Modified Harvard step test |
| [133] | To evaluate the validity of the Harvard step test for assessing physical fitness in Indian adolescent boys | 54 | 13 - 19 | Male = 54 | Harvard step test |
| [134] | To validate a new step test to predict VO_2max_ | NA | NA | NA | New step test |
| [135] | To evaluate two stepping procedures with an instrument and to develop an equation for predicting the oxygen cost | 60 | 29 - 68 | Male = 60 | Gradational step test |
| [136] | To share additional insights and findings regarding the use of the augmented two-step test | NA | NA | NA | Master two-step test |
| [137] | To discuss and address concerns related to the occurrence of chest or arm discomfort experienced during or after the master two-step test | 300 | NA | NA | Master two-step test |
| [138] | To investigate the occurrence and nature of pain experienced during the master two-step test, with a specific focus on identifying signs of impending angina | 300 | NA | NA | Master two-step test |
| [139] | To evaluate the practicality and consistency of the modified incremental step test for use in pulmonary rehabilitation | NA | NA | NA | Modified incremental step test |
| [140] | To identify the highest rate attained in the master two step test to demonstrate significant ischemic | 1 | 39 | Male = 1 | Master two-step test |
| [141] | To assess the effectiveness of various drug therapies, including vasodilators, amine oxidase inhibitors, and hypercholesteremic agents, in angina pectoris using the master two-step test | NA | NA | NA | Master two-step test |
| [142] | To compare different methods of estimation of VO_2max_ and heart rate recovery and evaluated the relationship of these estimates with cardiovascular risk factors. | 2286 | 35.7 ± 12 | Male = 1086 Female = 1100 | Tecumseh sub-maximal step test |
| [143] | To develop a new step test protocol to estimate maximal oxygen consumption measured during and after exercise | 192 | DG: 44.9 ± 7.6  VG: 49.8 ± 5.5 | Male = 103 Female = 89 | Japan step test |
| [144] | To compare the ergometric multistage exercise step test with the master double two-step test | NA | NA | NA | The master double 2-step test |
| [145] | To determine the energy expenditure associated with performing the master two-step test | 197 | 48.2 ± 5.7 | Male = 126 Female = 71 | Master two-step test |
| [146] | To compare the electrocardiogram recorded during exercise using radio electrocardiography with the postexercise ecg obtained from the master two-step test | 127 | NA | NA | Master two-step test |
| [147] | To evaluate the effects of circuit training on the modified Harvard step test | 34 | NA | NA | Modified Harvard step test |
| [148] | To determine whether the angle of the knee joint influences the fitness index scores of the Harvard step test | 33 | 20.03 ± 2.08 | Male = 33 | Harvard step test |
| [149] | To assess changes in exercise tolerance after an inpatient course of intravenous antibiotics, using the 3-min step test | 36 | 6 - 18 | Male = 20 Female = 16 | Three minute step test |
| [150] | To assess changes in exercise tolerance after an inpatient course of intravenous antibiotics, in children with cystic fibrosis | NA | NA | NA | Three minute step test |
| [151] | To develop a simple step test for measuring exercise systolic bp suitable for the routine clinic setting | 25 | NA | NA | Dundee step test |
| [152] | To evaluate the prognostic value of the Dundee step test in the management of hypertension | 2000 | 40 - 59 | Male = 2000 | Dundee step test |
| [153] | To compare adolescents with asthma and fixed airflow obstruction and adolescents with asthma without fixed airflow obstruction in terms of aerobic fitness | 44 | 15.6 ± 1.8 | Male = 25 Female = 19 | Incremental step test |
| [154] | To evaluate the effectiveness of the master two-step test in diagnosing coronary artery disease | NA | NA | NA | Master two-step test |
| [155] | To develop an incremental maximal step test to assess exercise capacity across the range of cystic fibrosis | 8 | 30.63 ± 5.89 | NA | Alfred step test exercise protocol |
| [156] | To estimate aerobic fitness among children, require substantial space and maximum effort | 170 | 7.7 ± 1.5 | Male = 87 Female = 83 | Simple effort independent step test |
| [157] | To investigate the relationship between oxygen desaturation during a 3-minute step test and long-term health outcomes in adults with cystic fibrosis | NA | NA | NA | Three-minute step test |
| [158] | To determine the feasibility and acceptability of the 3-minute step test as a test of exercise capacity in adults with cystic fibrosis | 101 | 29 ± 9 | Male= 56 Female = 45 | Three-minute step test |
| [159] | To evaluate the applicability of the two-minute step test in an assessment of exercise tolerance in patients with heart failure | 168 | NA | NA | The two-minute step test |
| [160] | To investigate the relationship between physical efficiency index calculated by the Harvard step test and heart rate variation | 16 | NA | Male = 8 Female = 6 | Harvard step test |
| [161] | To develop a submaximal cardiovascular fitness test | 75 | 19 - 56 | Male = 75 | Submaximal step test |
| [162] | To evaluate the construct validity and reproducibility of the 6-minute step test in individuals with obstructive sleep apnea treated with continuous positive airway pressure | 48 | 48 - 63 | NA | 6-minute step test |
| [163] | To compare the results of a load graded to maximum using both the step test and bicycle ergometer | 18 | 20 - 39 | Male = 18 | Graded step test |
| [164] | To cross-validate the Danish step test against an indirect maximal test, the watt-max test | 795 | 46.8 ± 8.2 | Male = 346 Female = 449 | Danish step test |
| [165] | To investigate the habitual smoking effects in heart rate response and heart rate recovery after step test in athletes | 78 | 27±8 | NA | 6-minute step test |
| [166] | To determine whether maximal strenuous exercise would produce an ischemic type of electrocardiographic change in young, normal subjects with a presumably normal coronary circulation | 210 | 17 - 65 | NA | Double two step test |
| [167] | To compare the exercise tolerance time, cardiopulmonary stress, and perception of effort between the Chester step test and a modified incremental step test | 32 | 50 - 87 | Male = 25 Female = 7 | Chester step test |
| [168] | To compare the physical exertion or workload between the multistage bicycle exercise test and the master’s two-step test | NA | NA | NA | Masters two-step test |
| [169] | To assess and compare a newly developed step test with a treadmill test in terms of evaluating cardiorespiratory working capacity | NA | NA | NA | New step test |
| [170] | To compare a body morphology index with two physical fitness tests in assessing physical working capacity | NA | NA | NA | Harvard step test |
| [171] | To validate the 3-minute step test and to compare 3-minute step test and 6-minute step test in stable patients with COPD as a tool to quantify the functional exercise capacity | 50 | 62.3 ± 7.9 | Male = 33 Female = 17 | Three-minute step test |
| [172] | To compare 3 different step tests in their ability to predict maximal oxygen consumption in young male subjects | NA | NA | Males | NA |
| [173] | To assess and compare the sensitivity of two diagnostic tests: master's double two-step test and a hypoxemia test, in individuals suspected of having coronary artery disease | NA | NA | NA | Master’s double step two test |
| [174] | To compare and evaluate the electrocardiographic changes induced by two diagnostic tests: master's double two-step test and levy's hypoxemia test | NA | NA | NA | Master’s double step two test |
| [175] | To investigate the effects of clofibrate, a lipid-lowering medication, on the results of the master 2-step test | NA | NA | NA | Master’s double step two test |
| [176] | To investigate whether Chester step test can differentiate functional capacity and the magnitude of cardiorespiratory response of patients with COPD from healthy subjects; and to compare it with the cardiorespiratory response induced by shuttle test and 6-minute walk test | 20 | 64±10 | Male = 6 Female = 14 | Chester step test |
| [177] | NA | NA | NA | NA | Chester step test |
| [178] | To determine the reliability of the Chester step test in patients with COPD and correlation with pulmonary function test and exercise test results | 32 | 69.9 ± 9.0 | Male = 29 Female = 3 | Chester step test |
| [179] | NA | NA | NA | NA | Chester step test |
| [180] | Efficient management using exercise programs with various benefits should be provided by educational institutions for children in their growth phase | 77 | 5 - 11 | Both | Harvard step test |
| [181] | NA | NA | NA | NA | 6-Minute step test |
| [182] | To verify whether heart rate is maintained below the calculated submaximal level in healthy, sedentary subjects when they perform the 6-minute step test and the 6-minute walking test, and to compare the maximal heart rate achieved by the subjects at the end of each test | 253 | 20 - 80 | Male = 108 Female = 145 | 6-Minute step test |
| [183] | To determine the effect of an exercise prescription instrument, compared to usual-care exercise counseling delivered by primary care doctors on fitness and exercise self-efficacy among elderly community-dwelling patients | 241 | > 65 | Male = 124 Female = 117 | The step test |
| [184] | To determine the therapeutic efficacy of rapid digitalization in acute myocardial infarction in the absence of apparent congestive heart failure | 800 | NA | NA | Master two step test |
| [185] | To investigate the relationship of body fat, blood coagulation time, and the Harvard step test recovery index | 54 | 21.6 ± 3.3 | Male | Harvard step test |
| [186] | To analyze the between-day reliability of this incremental step test in stable COPD patients | 18 | 68.3 ± 6.6 | NA | Incremental step-test |
| [187] | NA | NA | NA | NA | 2-min step test |
| [187] | NA | NA | NA | NA | 2-min step test |
| [188] | To identify whether the kasch pulse recovery test is a feasible tool for determining physiological readiness to engage in graded exercise, as an adjunct to standard testing and clinical judgment | 45 | 13.2 ± 2.1 | Male = 34  Female = 11 | 3-min step test |
| [189] | To determine the association of Japan step test-evaluated cardiovascular fitness with the prevalence of cardiovascular disease risk among Japanese workers | 885 | 30 - 60 | Male = 474 Female = 411 | Japan step test |
| [190] | To assess the physical fitness standards of Sedentary and physically active college students of eastern India in comparison to the young men of other countries | 352 | 16.0 - 24.5 | Male = 352 | Harvard step test |
| [191] | To assess cardiorespiratory fitness in children and adolescents with overweight/obesity using the kasch pulse recovery test, and its correlation with severity of obesity, insulin resistance, and blood pressure | 155 | 11.6 ± 1.8 | Both | 3-min step test |
| [192] | To investigate arrhythmias in the two-minute step test monitored and after exercise | NA | NA | NA | 2-min step test |
| [193] | To investigate arrhythmias in the two-minute step test | 600 | NA | NA | 2-min step test |
| [194] | To investigate arrhythmias in the two-minute step test | NA | NA | NA | 2-min step test |
| [195] | To compare the physiological response with a standardized fixed-rate step test with maximal cardiopulmonary exercise testing | 113 | 45 ± 13 | Male = 60 Female = 53 | Fixed-rate step test |
| [196] | To investigate the reliability and responsiveness, and the utility of the mist for exercise prescription in people with stable chronic lung disease | 40 | 72 ± 9 | Male = 17 Female = 23 | Modified incremental step test |
| [197] | To evaluate the applicability of the 4-minute step test, correlating its results with those of other measures and tests used with people with cardiovascular disease symptoms | 47 | 54 ± 12 | Female = 47 | 4-min step test |
| [198] | To determine the exercise counseling habits among a large group of Canadian family physicians and acceptance and utilization of an exercise counseling instrument geared to primary care practice | 400 | ≥ 65 | Both | NA |
| [199] | To estimate VO_2max_ using the step test index combined with heart rate variability in women with drug use disorder | 40 | G1 = 26.35 ± 4.45  G2 = 31.50 ± 4.11 | Female = 40 | 3-min step test |
| [200] | To improve the estimation of aerobic capacity through step tests by adjusting the step height to the individual's stature height | 100 | 19-35 | Male = 53 Female = 47 | Single stage step test |
| [201] | To elaborate a model predicting peak oxygen consumption in lean and obese children with use of the submaximal Chester step test | 169 | 11.5 ± 2.0 | Male = 78 Female = 91 | Adapted chester step test |
| [202] | To assess whether an adaptation of the Chester step test (with a progressive profile) can have maximal response characteristics in young women and compare it to the incremental shuttle walk test | 25 | 20.3 ± 1.5 | Female = 25 | Adapted chester step test |
| [203] | To compare maximal physiological responses between an incremental step test and cardiopulmonary exercise testing, to test the reproducibility of the incremental step test on different days, and to provide a predict equation to estimate VO_2_ from the incremental step test in patients with COPD | 34 | 67 ± 9 | Males = 34 | Incremental step-test |
| [204] | To compare maximal physiological responses between an Incremental Step test and cardiopulmonary exercise testing | 34 | NA | NA | Incremental step test |
| [205] | To determine whether a single-stage, self-paced, 6-minute step test provides reliable and reproducible estimates | 31 | 34-79 | Male = 19 Female = 12 | 6-min step test |
| [206] | To determine if a step test could be feasible, reliable, and valid for youths with educable or trainable mental retardation | 24 | 14.7 ± 2.7 | Male = 13 Female = 11 | Modification of the 3-min step test |
| [207] | To develop and validate a 6-minute step test protocol aimed at obtaining consistent and reproducible dyspnea responses in patients with COPD | 16 | 64.3 ± 8.1 | Male = 16 Female = 2 | 6-min step test |
| [208] | To investigate whether 4-minute step test would be a suitable replacement for 6-minute step test, in a simplified bode index, to predict mortality in COPD patients | 190 | 65 ± 9 | Male = 114 Female = 76 | 4-minute step test |
| [209] | To compare the step test results with those obtained from a modified astrand-rhyming cycle ergometer test to ensure the validity and reliability of the new step test protocol for use in population fitness testing | 48 | 37 ± 13 | Male = 19 Female = 29 | NA |
| [210] | To determine if the new height-adjustable step test model could be applied to children 6 to 18 years of age | 286 | 6 - 18 | Male = 146 Female = 140 | Harvard step test |
| [211] | To develop a simple height and rate specific step-test for children and adolescents | NA | NA | NA | Harvard step test |
| [212] | To study the potential usefulness of a sub-maximal self-paced step test as a prediction of maximal aerobic capacity in older adults in the primary care setting | 240 | ≥ 65 | Male = 118 Female = 122 | Self-paced step test |
| [213] | To introduce and validate a new single-stage step test that uses a height-adjustable platform to normalize the height of stepping for individuals of varying statures | 96 | 20.3 | Male = 48 Female = 48 | Single-stage step test |
| [214] | To introduce and evaluate a new single-stage step test designed for the clinical assessment of maximal oxygen consumption | 1 | 16 | Male = 1 | Single-stage step test |
| [215] | To predict maximal oxygen consumption using a new progressive step test | NA | NA | NA | New progressive step test |
| [216] | To assess whether a modification of the queen's college step test using a bench height based on knee joint angle of 90" may more accurately predict aerobic capacity than the original queen’s college step test | 18 | 18 - 37 | Female = 18 | Queens college step test |
| [217] | To develop a modified Harvard step test for women | 77 | 17 - 27 | Male = 46 Female = 31 | Modified Harvard step test |
| [218] | To validate a model reported to standardize the height of the stepping for individuals of various heights in males | 33 | 28 | Male = 33 | Three rate-specific step tests |
| [219] | To develop an equation to reduce variability of VO_2peak_ prediction from a step test and compare VO_2peak_ prediction from the new equation to the queen's college step test | 185 | 21.7 ± 2 | Male = 87 Female = 98 | Queen's college step test |
| [220] | To describe and compare the exercise intensity elicited by both high and low impact dance styles; To compare the relation between oxygen consumption and heart rate during a standard graded step test with that during different styles of dance and to evaluate the effect of additional arm work | 10 | 34.2 ± 4.6 | Female = 10 | Graded step test |
| [221] | To compare loads using step-test and ergometer | NA | NA | NA | NA |
| [222] | To develop norms and refine procedure for the astrand-ryhming step test to obtain accurate cardiorespiratory assessments of entering first-year students | 6688 | Male = 18.0 ± 0.65  Female = 17.9 ± 0.53 | Male = 5287 Female = 1401 | Astrand-ryhming step test |
| [223] | To compare the 3-minute step test with treadmill exercise for evaluating exercise-induced asthma in asthmatic children and assess whether responses to both tests are influenced by baseline lung function and habitual physical activity | 154 | 12.9 ± 0.9 | Male = 154 | 3-min step test |
| [224] | To correlate two-step test results with findings years later at death | NA | NA | NA | 2-minute step test |
| [225] | To indicate that "the two-step exercise test must not be considered an innocuous procedure, despite the absence of deleterious reports” | NA | NA | NA | 2-minute step test |

Note: HRR: Heart rate recovery; BMI: Body mass index; WC: waist circumference, WHtR: waist to height ratio; SRCT: Sitting and rising chair step test; SST: Stair Step Test; SMWT: Six minute Walk test; TMST: Two minute step test; COPD: chronic obstructive pulmonary disease ; YMCA: Young Men’s Christian Association; HBC: Heart Beat Count; DG: Derivation Group; VG: Validation Group; CG: Control Group; EG: Experimental Group; NA: Non Available; M: Mean; SD: Standard Deviation.

References

1. Matthews El, Horvat Fm, Phillips Da. Virtual Cardiorespiratory Fitness Testing During The Covid-19 Pandemic Using A Variable Height Step Test. Med Sci Sports Exerc. 2021; 53:3–4.

2. Matthews El, Horvat Fm, Phillips Da. Variable Height Step Test Provides Reliable Heart Rate Values During Virtual Cardiorespiratory Fitness Testing. Measurement In Physical Education And Exercise Science. 2022;26:155–64.

3. Cosby R, Mayo M. Value Of The Master 2-Step Test In Coronary Artery Disease. Am J Cardiol. 1959;3:444–8.

4. Knowlan D, Ardam I, Weinmann R. Value Of Radioelectrocardiography During Double Master 2-Step Test Compared With Standard Post-Exercise Electrocardiogram. Aerospace Medicine. 1964;35:273-.

5. Berlanga La, Matos-Duarte M, Abdalla P, Alves E, Mota J, Bohn L. Validity Of The Two-Minute Step Test For Healthy Older Adults. Geriatr Nurs. 2023;51:415–21.

6. Vidoni Ed, Mattlage A, Mahnken J, Burns Jm, Mcdonough J, Billinger Sa. Validity Of The Step Test For Exercise Prescription: No Extension To A Larger Age Range. J Aging Phys Act. 2013;21:444–54.

7. Pessoa Bv, Arcuri Jf, Labadessa Ig, Costa Jnf, Sentanin Ac, Di Lorenzo Vap. Validity Of The Six-Minute Step Test Of Free Cadence In Patients With Chronic Obstructive Pulmonary Disease. Rev Bras Fisioter. 2014;18:228–36.

8. Chen S-M, Wang J-S, Lee W-C, Hou C-W, Chen C-Y, Laio Y-H, Et Al. Validity Of The 3 Min Step Test In Moderate Altitude: Environmental Temperature As A Confounder. Appl Physiol Nutr Metab. 2006;31:726–30.

9. Bennett H, Parfitt G, Davison K, Eston R. Validity Of Submaximal Step Tests To Estimate Maximal Oxygen Uptake In Healthy Adults. Sports Medicine. 2016;46:737–50.

10. Chatterjee S, Chatterjee P, Mukherjee Ps, Bandyopadhyay A. Validity Of Queen’s College Step Test For Use With Young Indian Men. Br J Sports Med. 2004;38:289–91.

11. Chatterjee S, Chatterjee P, Bandyopadhyay A. Validity Of Queen’s College Step Test For Estimation Of Maximum Oxygen Uptake In Female Students. Indian J Med Res. 2005;121:32–5.

12. Bennett H, Davison K, Parfitt G, Eston R. Validity Of A Perceptually-Regulated Step Test Protocol For Assessing Cardiorespiratory Fitness In Healthy Adults. Eur J Appl Physiol. 2016;116:2337–44.

13. Cooney Jk, Ahmad Ya, Moore Jp, Lemmey Ab, Jones Jg, Madisson Pj, Et Al. Validity And Reliability Of The Siconolfi Step Test For Estimating Cardio-Respiratory Fitness In Rheumatoid Arthritis Patients. Rheumatology. 2011;50:84–84.

14. Marcora Sm, Casanova F, Fortes Mb, Maddison Pj. Validity And Reliability Of The Siconolfi Step Test For Assessment Of Physical Fitness In Patients With Systemic Lupus Erythematosus. Arthritis Rheum-Arthritis Care Res. 2007;57:1007–11.

15. Arcuri Jf, Borghi-Silva A, Labadessa Ig, Sentanin Ac, Candolo C, Pires Di Lorenzo Va. Validity And Reliability Of The 6-Minute Step Test In Healthy Individuals: A Cross-Sectional Study. Clinical Journal Of Sport Medicine. 2016;26:69–75.

16. Travensolo C De F, Arcuri Jf, Polito Md. Validity And Reliability Of The 6-Min Step Test In Individuals With Coronary Artery Disease. Physiother Res Int. 2020;25:E1810.

17. Vilarinho R, Serra L, Aguas A, Alves C, Silva Pm, Caneiras C, Et Al. Validity And Reliability Of A New Incremental Step Test For People With Chronic Obstructive Pulmonary Disease. Bmj Open Respir Res. 2022;9:E001158.

18. Knight E, Stuckey Mi, Petrella Rj. Validation Of The Step Test And Exercise Prescription Tool For Adults. Canadian Journal Of Diabetes. 2014;38:164–71.

19. Kulberg J, Kasch F. Validation Of The Kasch 3 Minute Step Test. Medicine And Science In Sports And Exercise. 1979;11:94.

20. Hong Sh, Yang Hi, Kim D-I, Gonzales Ti, Brage S, Jeon Jy. Validation Of Submaximal Step Tests And The 6-Min Walk Test For Predicting Maximal Oxygen Consumption In Young And Healthy Participants. Ijerph. 2019;16:4858.

21. Bragada Ja, Bartolomeu Rf, Rodrigues Pm, Magalhães Pm, Bragada Jp, Morais Je. Validation Of Steptest4all For Assessing Cardiovascular Capacity In Young Adults. International Journal Of Environmental Research And Public Health. 2022;19:11274.

22. Harkrider Tl, Hill Dw. Validation Of A Step Test To Predict Vo(2max). Medicine And Science In Sports And Exercise. 2006;38:S507.

23. Hansen D, Jacobs N, Thijs H, Dendale P, Claes N. Validation Of A Single-Stage Fixed-Rate Step Test For The Prediction Of Maximal Oxygen Uptake In Healthy Adults. Clin Physiol Funct Imaging. 2016;36:401–6.

24. Gossett T, Shepherd T, Powell R, Mehta S. Validation Of A Multi-Staged Step Test For Measuring Fitness As A Clinical Vital Sign. Med Sci Sports Exerc. 2017;49:299–299.

25. Beutner F, Ubrich R, Zachariae S, Engel C, Sandri M, Teren A, Et Al. Validation Of A Brief Step-Test Protocol For Estimation Of Peak Oxygen Uptake. European Journal Of Preventive Cardiology. 2015;22:503–12.

26. Francis K, Cuipepper M. Validation Of A 3 Minute Hight-Adjusted Step Test. Journal Of Sports Medicine And Physical Fitness. 1988;28:229–33.

27. Reychler G, Audag N, Dewulf S, Mestre Nm, Caty G. Validation Of 6 Min Step Test And 4-M Gait Speed In Children: A Randomized Cross-Over Study. Gait Posture. 2018;61:19–24.

28. Kulbertus H, Humblet L. Transient Hemiblock - Abnormal Type Of Response To Master 2-Step Test. Am Heart J. 1972;83:574-+.

29. Zaidi S, Rylance J, Roberts S, Finnerty J, Benton I, Ponnuswamy A, Et Al. To Ascertain Usefulness Of Chester Step Test In Pulmonary Rehabilitation Programmes. Eur Resp J. 2013;42.

30. Narang I, Pike S, Rosenthal M, Balfour-Lynn Im, Bush A. Three-Minute Step Test To Assess Exercise Capacity In Children With Cystic Fibrosis With Mild Lung Disease. Pediatr Pulmonol. 2003;35:108–13.

31. Borel B, Wilkinson-Maitland Ca, Hamilton A, Bourbeau J, Perrault H, Jensen D, Et Al. Three-Minute Constant Rate Step Test For Detecting Exertional Dyspnea Relief After Bronchodilation In Copd. Int J Chronic Obstr Pulm Dis. 2016;11:2991–3000.

32. Francis K, Culpepper M. The Validation Of A Height Adjusted, Rate Specific Single-Stage Step Test. Physical Therapy. 1988;68:856.

33. Silva De Andrade Cf, Cianci Rg, Malaguti C, Dal Corso S. The Use Of Step Tests For The Assessment Of Exercise Capacity In Healthy Subjects And In Patients With Chronic Lung Disease. J Bras Pneumol. 2012;38:116–24.

34. Teixeira Jb, Ferreira Stbp, Bernardino Mec, Magalhaes Mgs, Bezerra P, De Lima Amj. The Three-Minute Step Test Showed Good Construct Validity And Intra-Rater Reliability In Healthy Children Aged 7-11 Years. Acta Paediatr. 2020;109:2354–5.

35. Thompson P, Caine R, Thom J, Law R, Ahmad Y, Lemmey A, Et Al. The Submaximal Siconolfi Step Test Reliably Estimates Cardiorespiratory Fitness Levels In Patients With Axial Spondyloarthropathy. Rheumatology. 2015;54:139–139.

36. Ritti-Dias Rm, Farah Bq. The Six-Minute Step Test As An Alternative For Functional Capacity Assessment In Patients With Cardiovascular Diseases. Arq Bras Cardiol. 2021;116:896–7.

37. Fonteles Ritt Le, Darze Es, Feitosa Gf, Porto Js, Bastos G, Linhares De Albuquerque Rb, Et Al. The Six-Minute Step Test As A Predictor Of Functional Capacity According To Peak Vo2 In Cardiac Patients. Arq Bras Cardiol. 2021;116:889–95.

38. Di Thommazo-Luporini L, Pinheiro Carvalho L, Luporini Rl, Trimer R, Falasco Pantoni Cb, Catai Am, Et Al. The Six-Minute Step Test As A Predictor Of Cardiorespiratory Fitness In Obese Women. Eur J Phys Rehabil Med. 2015;51:793–802.

39. Silva Km, Raphael Y, Parnayba J, Da Cal M, Figueira B, Condesso D, Et Al. The Six - Minute Step Test Standard Equation For Sedentary Subjects. Am J Respir Crit Care Med. 2014;189.

40. Lemanska A, Poole K, Aning Jj, Griffin Ba, Manders R, Saxton Jm, Et Al. The Siconolfi Step Test: A Valid And Reliable Assessment Of Cardiopulmonary Fitness In Older Men With Prostate Cancer. Eur Rev Aging Phys Act. 2019;16:1.

41. Gouteron A, Besson D, Gudjoncik A, Hannequin A, Laroche D, Casillas J-M. The Short And Fast Step Test: A Functional Tool To Assess Anaerobic Metabolism In Rehabilitated Coronary Patients. Ann Phys Rehabil Med. 2020;63:368–71.

42. Lin C-H, Kung H-W, Lin F-C, Wu M-C, Kuo C-H. The Relation Between Metabolism Fitness And Step-Test Index In Different Ranges Of Age. Med Sci Sports Exerc. 2006;38:S508–S508.

43. Da Silva Km, De Souza Y, Parnayba J, Maiworm A, Da Cal M, Figueira B, Et Al. The Reference Equation To Six-Minute Step Test: A Benchmark To Evaluate The Functional Capacity In Patients. Am J Respir Crit Care Med. 2016;193.

44. Srithawong A, Poncumhak P, Manoy P, Kumfu S, Promsrisuk T, Prasertsri P, Et Al. The Optimal Cutoff Score Of The 2-Min Step Test And Its Association With Physical Fitness In Type 2 Diabetes Mellitus. J Exerc Rehabil. 2022;18:214–21.

45. Bales Dw, Craig Bn, Congleton Jj, Kerk Cj, Amendola Aa, Gaines Wg, Et Al. The Influence Of Supporting The Oxylog Instrument On Estimated Maximal Aerobic Capacity During A Step Test And Heart Rate In A Lifting Test. Appl Ergon. 2001;32:367–77.

46. Campbell W. The Harvard Step Test. Br Med J. 1958;1:1179–1179.

47. Unterman D, Degraff A. The Effect Of Exercise On The Electrocardiogram (Master 2-Step Test) In The Diagnosis Of Coronary Insufficiency. Am J Med Sci. 1948;215:671–85.

48. Elliott D, Abt G, Barry T. The Effect Of An Active Arm Action On Heart Rate And Predicted Vo2ma During The Chester Step Test. J Sci Med Sport. 2008;11:112–5.

49. Lim Po, Shiels P, Macdonald Tm. The Dundee Step Test: A Novel Exercise Test Suitable For The Outpatient Management Of Hypertension. J Hypertens. 1998;16:1701–1701.

50. Abu Hanifah R, Mohamed Mna, Jaafar Z, Mohsein Na-Sa, Jalaludin My, Majid Ha, Et Al. The Correlates Of Body Composition With Heart Rate Recovery After Step Test: An Exploratory Study Of Malaysian Adolescents. Plos One. 2013;8:E82893.

51. Izquierdo Mc, Lopes S, Teixeira M, Polonia J, Alves Aj, Mesquita-Bastos J, Et Al. The Chester Step Test Is A Valid Tool To Assess Cardiorespiratory Fitness In Adults With Hypertension: Reducing The Gap Between Clinical Practice And Fitness Assessments. Hypertens Res. 2019;42:2021–4.

52. Peroy-Badal R, Sevillano-Castano A, Nunez-Cortes R, Garcia-Fernandez P, Torres-Castro R, Vilaro J, Et Al. The Chester Step Test Is A Reproducible Tool To Assess Exercise Capacity And Exertional Desaturation In Post-Covid-19 Patients. Healthcare. 2023;11:51.

53. Planner S, Morrison L. The Chester Step Test - Is This A Valid Predictor Of Disease Severity In Adult Cf? Pediatr Pulmonol. 2007;360–360.

54. Barron I, Quintana Riera S, Reychler G. The 3 Minute Step Test Is A Validated Field Test To Evaluate The Functional Exercise Capacity In Children Aged 6 To 12. Respir Med Res. 2021;80:100833.

55. Master A, Pordy L, Arai H, Field L, Storch S. The 2-Step Test In The Diagnosis Of Coronary Insufficiency. New York State Journal Of Medicine. 1950;50:821–821.

56. Sevillano-Castano Ai, Peroy-Badal R, Torres-Castro R, Canuelo-Marquez Am, Rozalen-Bustin M, Modrego-Navarro A, Et Al. Test-Retest Reliability And Minimal Detectable Change In Chester Step Test And 1-Minute Sit-To-Stand Test In Long Covid Patients. Appl Sci-Basel. 2023;13:8464.

57. Ganeriwal Sk, Sen Sc, Khandare Ss. Test Of Physical Fitness (Harvard Step Test) In Indian Females. Indian J Med Res. 1968;56:845–9.

58. Ghosh A, Pan R. Systolic And Diastolic Recovery Following The Harvard Step Test In Adult Asian Indian Men. Anthropol Anz. 2007;65:427–34.

59. Dal Corso S, Oliveira An, Izbicki M, Ciani Rg, Malaguti C, Nery Le. Symptom-Limited Incremental Step Test In Copd Patients: Reproducibility And Validity Compared To Incremental Cycle Ergometry. Am J Respir Crit Care Med. 2009;179.

60. Starkoff Be, Cuttler L, Uli N, Mcaleer S, Schmidt A, Ievers-Landis Ce, Et Al. Submaximal Step-Testing As A Tool To Assess Cardiovascular Fitness In Youth Who Are Obese. Med Sci Sports Exerc. 2011;43:892–892.

61. Eston R, Bennett H, Parfitt G, Davison K. Author’s Reply To Sabour And Ghassemi "Submaximal Step Tests To Estimate Maximal Oxygen Uptake In Healthy Adults: Methodological Issues About Validity And Reliability’’. Sports Med. 2016;46:1383–4.

62. Shukuya M, Yoshida S, Nakayama A, Ozawa S, Saito T, Masuda Y, Et Al. Study On Equivalent Work Load Of Bicycle Ergometer With Masters 2-Step Test. Jpn Circ J-Engl Ed. 1973;37:903–4.

63. Jose A, Dal Corso S. Step Tests Are Safe For Assessing Functional Capacity In Patients Hospitalized With Acute Lung Diseases. J Cardiopulm Rehabil Prev. 2016;36:56–61.

64. Day J. Statistical Investigation Of Ryhming Step Test. Research Quarterly. 1967;38:539–43.

65. Fonteles Ritt Le, Porto J, Claro T, Cavalcante D, Feitosa Cm, Prado E, Et Al. Stair Step Test And Sitting Rising Chair Test As Predictors Of Maximal Oxigen Uptake. J Am Coll Cardiol. 2017;69:1733–1733.

66. Cogswell R, Henderson C, Berryman G, Harris S, Ivy A, Youmans J. Some Observations Of The Effects Of Training On Pulse Rate, Blood Pressure And Endurance, In Humans, Using The Step Test (Harvard), Treadmill And Electrodynamic Brake Bicycle Ergometer. Am J Physiol. 1946;146:422–30.

67. Bohannon Rw, Bubela Dj, Wang Y-C, Magasi Ss, Gershon Rc. Six-Minute Walk Test Vs. Three-Minute Step Test For Measuring Functional Endurance. J Strength Cond Res. 2015;29:3240–4.

68. Maynard K, Maiworm A, Raphael Y, Da Cal M, Figueira B, Condesso D, Et Al. Six-Minute Step Test Or Six-Minute Walk Test In Healthy And Sedentary Individuals: Are They Both Safe Tests? Am J Respir Crit Care Med. 2013;187.

69. Da Silva Km, Raphael Y, Condesso D, Figueira B, Maiworm A, Da Cal M, Et Al. Six-Minute Step Test In Healthy And Sedentary Individuals: Is It A Safe Test? Eur Resp J. 2014;44.

70. Arcuri Jf, Ferreira Da Costa Jn, Labadessa Ig, Pessoa Bv, Jamami M, Pires Di Lorenzo Va. Six Minute Step Test Reproducibility In Copd Patients: Cross-Sectional Study. Eur Resp J. 2013;42.

71. Suzuki K, Akashi Yj, Manabe M, Mizukoshi K, Kamijima R, Kou S, Et Al. Simple Exercise Echocardiography Using A Master’s Two-Step Test For Early Detection Of Pulmonary Arterial Hypertension. J Cardiol. 2013;62:176–82.

72. Jelinek V, Herman M, Schweitzer P, Gorlin R. Significance Of Chest Pain Occurring With Master 2 Step Test. Aust N Z J Med. 1976;6:22–5.

73. Russek H. Serum Cholesterol And Master 2-Step Test Responses In Coronary Patients Receiving Hypocholesteremic Agents - Thyroid Extract, Dextrothyroxine, Dextrotriiodothyronine, Linoleic And Arachidonic Acids, And Triparanol. Circulation. 1961;24:1027-.

74. Master A. S-T Elevations In Master 2-Step Test - Frequency And Significance. Circulation. 1973;48:249–249.

75. Bigard A. Review Of 2 Years Routine Use Of The Step Test For The Indirect Determination Of Maximal Oxygen-Consumption In The 27th Alpine Battalion. Medecine Et Armees. 1983;11:689–95.

76. Skuratova N, Belyaeva L. Results Of Harward Step-Test On Young Athletes. J Cardiovasc Electrophysiol. 2011;22:S100–S100.

77. Davi Sf, Arcuri Jf, Labadessa Ig, Pessoa Bv, Ferreira Da Costa Jn, Sentanin Ac, Et Al. Reproducibility Of The Six-Minute Walk And Step Tests In Healthy Young Adults. Rev Bras Med Esporte. 2014;20:214–8.

78. Munari Ab, Cristiana Santos Silva Ij, Gulart Aa, Venancio Rs, Klein Sr, Zanotto J, Et Al. Reproducibility Of The 6-Min Step Test In Subjects With Copd. Respir Care. 2021;66:292–9.

79. Camargo Aa, Lanza Fc, Tupinamba T, Corso Sd. Reproducibility Of Step Tests In Patients With Bronchiectasis. Braz J Phys Ther. 2013;17:255–62.

80. Da Costa Jnf, Arcuri Jf, Goncalves Il, Davi Sf, Pessoa Bv, Jamami M, Et Al. Reproducibility Of Cadence-Free 6-Minute Step Test In Subjects With Copd. Respir Care. 2014;59:538–42.

81. Master A. Reminiscences Of 50 Years In Cardiology At Mount-Sinai With Special Reference To 2-Step Test. Mt Sinai J Med. 1972;39:486-.

82. Ng Rs, Lin Vfp. Reliability Of 3-Min Step Test Using 2 Stepping Cadences In Hong Kong University Students. Med Sci Sports Exerc. 2008;40:S420–S420.

83. Alves A, Oliveira A, Ferreira Pg, Marques A. Reliability And Validity Of The Chester Step Test In People With Interstitial Lung Disease. Eur Resp J. 2021;58.

84. Giacomantonio N, Morrison P, Rasmussen R, Mackay-Lyons Mj. Reliability And Validity Of The 6-Minute Step Test For Clinical Assessment Of Cardiorespiratory Fitness In People At Risk Of Cardiovascular Disease. Journal Of Strength And Conditioning Research. 2020;34:1376–82.

85. Marinho Rs, Jurgensen Sp, Arcuri Jf, Goulart Cl, Dos Santos Pb, Roscani Mg, Et Al. Reliability And Validity Of Six-Minute Step Test In Patients With Heart Failure. Brazilian J Med Biol Res. 2021;54:E10514.

86. Buckley Jp, Sim J, Eston Rg, Hession R, Fox R. Reliability And Validity Of Measures Taken During The Chester Step Test To Predict Aerobic Power And To Prescribe Aerobic Exercise. Br J Sports Med. 2004;38:197–205.

87. Chung Mml, Chan Rwy, Fung Y-K, Fong Ssm, Lam Ssl, Lai Cwk, Et Al. Reliability And Validity Of Alternate Step Test Times In Subjects With Chronic Stroke. J Rehabil Med. 2014;46:969–74.

88. Mcardle W, Katch F, Pechar G, Jacobson L, Ruck S. Reliability And Interrelationships Between Maximal Oxygen Intake, Physical Work Capacity And Step-Test Scores In College Women. Medicine And Science In Sports. 1972;4:182–6.

89. Fagundes Cf, Di Thommazo-Luporini L, Goulart Cl, Braatz D, Ditomaso A, Borghi-Silva A. Reference Equations Of Oxygen Uptake For The Step Test In The Obese Population. Brazilian J Med Biol Res. 2022;55:E11864.

90. Vilarinho R, Toledo A, Silva C, Melo F, Tomaz L, Martins L, Et Al. Reference Equation Of A New Incremental Step Test To Assess Exercise Capacity In The Portuguese Adult Population. Jcm. 2022;12:271.

91. Rosenfeld I, Rosenfeld C, Master A. Recording Electrocardiogram During Performance Of Master 2-Step Test .I. Circulation. 1964;29:204-+.

92. Galvis-Rincon Jc, Mejia-Cano Je, Espinosa-Delaossa Pj. Queen’s College Step Test Correlation And Ergoespirometry For Vo_2_max Estimation. Rev Iberoam Cienc Act Fis Deport. 2020;9:94–107.

93. Shiono S, Endo M, Nakahashi K, Nakatsuka M. Preoperative Master’s Double Two-Step Test May Predict Survival After Lobectomy In Patients With Lung Cancer. J Cardiothorac Surg. 2022;17:91.

94. Jacks D, Moore Jb, Topp R, Bibeau Ws. Prediction Of Vo2 Peak Using A Sub-Maximal Bench Step Test In Children. Med Sci Sports Exerc. 2008;40:S418–S418.

95. Lee O, Lee S, Kang M, Mun J, Chung J. Prediction Of Maximal Oxygen Consumption Using The Young Men’s Christian Association-Step Test In Korean Adults. Eur J Appl Physiol. 2019;119:1245–52.

96. Shephard R. Prediction Of Maximal Oxygen Consumption Using A New Progressive Step Test. Ergonomics. 1967;10:1+.

97. Carvalho Lp, Di Thommazo-Luporini L, Aubertin-Leheudre M, Bonjorno Junior Jc, De Oliveira Cr, Luporini Rl, Et Al. Prediction Of Cardiorespiratory Fitness By The Six-Minute Step Test And Its Association With Muscle Strength And Power In Sedentary Obese And Lean Young Women: A Cross-Sectional Study. Plos One. 2015;10:E0145960.

98. Santo As, Golding La. Predicting Maximum Oxygen Uptake From A Modified 3-Minute Step Test. Research Quarterly For Exercise And Sport. 2003;74:110–5.

99. Munari Ab, Venancio Rs, Klein Sr, Gulart Aa, Silva Ijcs, Sonza A, Et Al. Physiological Responses To The 6-Min Step Test In Patients With Chronic Obstructive Pulmonary Disease. J Cardiopulm Rehabil Prev. 2020;40:55–61.

100. Hellerstein H, Ford A, Littman W. Physiologic Response To The Master 2-Step Test In Normotensive Patients With Coronary Disease. Circulation. 1955;12:721–721.

101. Ford A, Hellerstein H, Littman W, Phillips J, Gousios A. Physiologic Response To The Master 2 Step Test In Normal Males. Circulation. 1955;12:705–705.

102. Rowell L, Taylor H, Simonson E, Carlson W. Physiologic Fallacy Of Adjusting For Body Weight In Performance Of Master 2-Step Test. Am Heart J. 1965;70:461-.

103. Buckley J, Sim J, Eston R, Hession R, Fox R. Perceived Exertion And Heart Rate Reliability During The Chester Step Test. J Sports Sci. 2003;21:263–4.

104. Master A, Rosenfel. I. Pain Patterns In 2-Step Test - Concept Of Impending Angina. Journal Of The American Medical Association. 1969;208:1473-.

105. Albuquerque Vs, Dal Corso S, Do Amaral Dp, Dutra De Oliveira Tm, Souza Gf, Silva De Souza Rn, Et Al. Normative Values And Reference Equation For The Six-Minute Step Test To Evaluate Functional Exercise Capacity: A Multicenter Study. J Bras Pneumol. 2022;48:E20210511.

106. Lim Po, Macdonald Tm. Non-Invasive Profiling Of Total Peripheral Vascular Resistance Using The Dundee Step Test. Am J Hypertens. 1999;12:173a-173a.

107. Grossman L, Grossman M. Myocardial Infarction Precipitated By Master 2-Step Test. Jama-J Am Med Assoc. 1955;158:179–80.

108. Mcnabb J. Myocardial Infarction And Master 2-Step Test. Jama-J Am Med Assoc. 1955;158:971–971.

109. Master A. Myocardial Infarction And Master 2-Step Test. Jama-J Am Med Assoc. 1955;158:970–970.

110. Master A, Rosenfeld I. Monitored + Post-Exercise 2-Step Test - Detection Of Silent Coronary Heart Disease + Differential Diagnosis Of Chest Pain. Jama-J Am Med Assoc. 1964;190:494-.

111. Cotten D. Modified Step Test For Group Cardiovascular Testing. Research Quarterly. 1971;42:91–5.

112. Coll F, Hill K, Burrows S, Watson C, Edgar D. Modified Chester Step Test In A Healthy Adult Population: Measurement Properties And Development Of A Regression Equation To Estimate Test Duration. Physical Therapy. 2020;100:1411–8.

113. Vieira Eb, Degani-Costa Lh, Amorim Bc, Oliveira Lb, Miranda-Silva T, Sperandio Pca, Et Al. Modified Bode Index To Predict Mortality In Individuals With Copd: The Role Of 4-Min Step Test. Respir Care. 2020;65:977–83.

114. Hygino J. Modeling The Heart Rate Response To Step Test. Med Sci Sports Exerc. 2004;36:S114–5.

115. Fukuda I, Yoshida T, Yoritsune H, Kiso T, Kitazawa A, Mozai T, Et Al. Masters Double 2 Step Test And Coopers Aerobic Programmed Treadmill Test In Patients With Cardiovascular Disorders. Jpn Circ J-Engl Ed. 1978;42:804–804.

116. Russek H. Master 2-Step Test In Coronary Artery Disease. Jama-J Am Med Assoc. 1957;165:1772–5.

117. Constant J. Master 2-Step Test - Present Status. New York State Journal Of Medicine. 1980;80:39–45.

118. Master A, Rosenfeld I. Master 2-Step Test - Evaluation Of Clinical Usefulness In 650 Persons Subjected To Extended Follow-Up Studies. Am J Cardiol. 1964;13:122-.

119. Master A. Master 2-Step Test. Am Heart J. 1968;75:809-.

120. Chrisoherakis G, Karabinos I, Papanikolaou N, Papagoras C, Kostaki P, Papadopoulos A, Et Al. Late-Breaking Abstract: Maximum Oxygen Uptake (Maxvo2) During A 6-Minute Step Test (6-Mst) In Healthy People: Validation Of A Low Cost Exercise Test. Eur Resp J. 2015;46.

121. Dourado Im, Santos Pb, Goulart Cl, Marinho Rs, Santos-De-Araujo Ad, Roscani Mg, Et Al. Is The Six-Minute Step Test Able To Reflect The Severity And Symptoms Based On Cat Score? Heart Lung. 2023;58:28–33.

122. Besson D, Gouteron A, Sinssaine-Ayillo A, Casillas J-M, Rigaud L, Ornetti P, Et Al. Is The Short And Fast Step Test A Safe And Feasible Tool For Exploring Anaerobic Capacities Of Individuals With Coronary Heart Disease In Clinical Practice? Eur J Phys Rehabil Med. 2021;57:977–84.

123. Buckley Jp, Sim J, Hession R, Fox R. Is The Error In Predicting *V*o_2max_ From The Chester Step Test More Related To Errors In Estimated Maximal Heart Rate Or Errors In The Estimated Oxygen Cost Of A Given Stepping Work Rate? J Sports Sci. 2002;20:17–17.

124. Master A. Is Highest Rate Attained In Master 2-Step Test Sufficient. Circulation. 1970;42:Ii19-+.

125. Teren A, Zachariae S, Beutner F, Ubrich R, Sandri M, Engel C, Et Al. Incremental Value Of Veterans Specific Activity Questionnaire And The Ymca-Step Test For The Assessment Of Cardiorespiratory Fitness In Population-Based Studies. Eur J Prev Cardiol. 2016;23:1221–7.

126. Vieira E, Ramos R, Ivanaga I, Rolim J, Fonseca A, Rodrigues Junior J, Et Al. Incremental Step Test In Patients With Pulmonary Hypertension. Eur Resp J. 2018;52.

127. Lim Po, Donnan Pt, Macdonald Tm. How Well Do Office And Exercise Blood Pressures Predict Sustained Hypertension? A Dundee Step Test Study. J Hum Hypertens. 2000;14:429–33.

128. Makous N, Cha H, Taylor E. Hemodynamics Of Master 2-Step Test In Hypertension + Healed Mycocardial Infarction. Circulation. 1964;30:77-.

129. Makous N, Taylor E, Decha H. Hemodynamic Changes Following Master 2-Step Test In Hypertension. Circulation. 1963;28:761-.

130. Francis K, Culpepper M. Height-Adjusted, Rate-Specific, Single-Stage Step Test For Predicting Maximal Oxygen Consumption: Southern Medical Journal. 1989;82:602–6.

131. Hillmervogel U, Langer H, Kanzow E. Heart-Rate, Pulmonary Ventilation And Arterial Lactate Concentration In Step-Tests. Int J Sports Med. 1987;8:132–3.

132. Montoye H, Willis P, Cunningham D, Keller J. Heart Rate Response To A Modified Harvard Step Test - Males And Females, Age 10-69. Research Quarterly. 1969;40:153–62.

133. Banerjee P, Chatterjee S. Harvard Step Test As A Measure Of Physical-Fitness In Adolescent Boys. Indian J Med Res. 1984;79:413–7.

134. Harkrider Tl, Hill Dw. Validation Of A Step Test To Predict Vo_2max_. Med Sci Sports Exerc. 2006;38:S507–S507.

135. Nagle F, Balke B, Naughton J. Gradational Step Tests For Assessing Work Capacity. J Appl Physiol. 1965;20:745-.

136. Master A. Further Experiences On Augmented 2-Step Test. Circulation. 1972;46:269-+.

137. Master A. Frequency And Time Of Occurrence Of Pain During Master 2-Step Test - Impending Angina. Chest. 1971;59:125-.

138. Master A. Frequency And Characteristics Of Pain During Master 2-Step Test - Impending Angina. Diseases Of The Chest. 1969;56:258-.

139. Burge A, Dal Corso S, Bondarenko J, Handley E, Maclachlan S, Abramson M, Et Al. Feasibility And Reproducibility Of The Modified Incremental Step Test For Pulmonary Rehabilitation. Respirology. 2019;24:65–65.

140. Master A. Fastest Rate Attained In Master 2-Step Test. Am Heart J. 1974;87:139–139.

141. Russek H. Evaluation Of Drug Therapy In Angina Pectoris Employing Master 2-Step Test - Vasodilators, Amine Oxidase Inhibitors, And Hypocholesteremic Agents. Circulation. 1961;24:1028-.

142. Hughes Ad, Chaturvedi N. Estimation Of Maximal Oxygen Consumption And Heart Rate Recovery Using The Tecumseh Sub-Maximal Step Test And Their Relationship To Cardiovascular Risk Factors. Artres. 2017;18:29.

143. Matsuo T, So R, Takahashi M. Estimating Cardiorespiratory Fitness From Heart Rates Both During And After Stepping Exercise: A Validated Simple And Safe Procedure For Step Tests At Worksites. Eur J Appl Physiol. 2020;120:2445–54.

144. Kurihara T, Usami M, Narita M. Ergometric Multistage Exercise Test - Comparison With Master Double 2-Step Test. Jpn Circ J-Engl Ed. 1975;39:1009–1009.

145. Ford A, Hellerstein H. Energy Cost Of The Master 2-Step Test. Jama-J Am Med Assoc. 1957;164:1868–74.

146. Bellet S, Eliakim M, Deliyiannis S. Electrocardiogram During Exercise As Recorded By Radioelectrocardiography - Comparison With Postexercise Electrocardiogram (Master 2-Step Test). Circulation. 1961;24:884-+.

147. Howell M, Hodgson J, Sorenson J. Effects Of Circuit Training On The Modified Harvard Step Test. Research Quarterly. 1963;34:154–7.

148. Ariel G. Effect Of Knee-Joint Angle On Harvard Step-Test Performance. Ergonomics. 1969;12:33-.

149. Pike Se, Prasad Sa, Balfour-Lynn Im. Effect Of Intravenous Antibiotics On Exercise Tolerance (3-Min Step Test) In Cystic Fibrosis. Pediatr Pulmonol. 2001;32:38–43.

150. Pike Se, Prasad Sa, Slade G, Rae J, Balfour-Lynn Im. Effect Of Intravenous Antibiotics (Ivabs) On Exercise Tolerance (3-Minute Step Test) In Children With Cf. Am J Respir Crit Care Med. 1999;159:A684–A684.

151. Lim Po, Shiels P, Anderson J, Macdonald Tm. Dundee Step Test: A Simple Method Of Measuring The Blood Pressure Response To Exercise. J Hum Hypertens. 1999;13:521–6.

152. Lim Po, Donnan Pt, Macdonald Tm. Does The Dundee Step Test Predict Outcome In Treated Hypertension? A Sub-Study Protocol For The Ascot Trial. J Hum Hypertens. 2000;14:75–8.

153. Barbosa R, Silva R, Camargo S, Dal Corso S, Salge Jm, Fonseca Aj, Et Al. Does Modified Incremental Step Test Evaluate Maximal Exercise Capacity In Moderate To Severe Subjects With Asthma? Eur Resp J. 2019;54.

154. Russek H. Diagnostic Value Of The Master 2-Step Test In Coronary Artery Disease. Circulation. 1958;18:774–774.

155. Wilson Lm, Ellis Mj, Lane Rl, Wilson Jw, Keating Dt, Jaberzadeh S, Et Al. Development Of The A-Step: A New Incremental Maximal Exercise Capacity Step Test In Cystic Fibrosis. Pediatric Pulmonology. 2021;56:3777–84.

156. Hayes Rm, Maldonado D, Gossett T, Shepherd T, Mehta Sp, Flesher Sl. Developing And Validating A Step Test Of Aerobic Fitness Among Elementary School Children. Physiother Can. 2019;71:187–94.

157. Button B, Rasekaba T, Wilson J, Holland Ae. Desaturation On 3-Minute Step Test Is Associated With Impaired Outcomes At 12 Months In Adults With Cf. Pediatr Pulmonol. 2009;366–366.

158. Holland Ae, Rasekaba T, Wilson Jw, Button Bm. Desaturation During The 3-Minute Step Test Predicts Impaired 12-Month Outcomes In Adult Patients With Cystic Fibrosis. Respiratory Care. 2011;56:1137–42.

159. Wegrzynowska-Teodorczyk K, Mozdzanowska D, Josiak K, Siennicka A, Nowakowska K, Banasiak W, Et Al. Could The Two-Minute Step Test Be An Alternative To The Six-Minute Walk Test For Patients With Systolic Heart Failure? Eur J Prev Cardiol. 2016;23:1307–13.

160. Kim D-H, Cho Y-H, Seo T-B. Correlation Between Physical Efficiency Index Using Harvard Step Test And Heart Rate Variation In College Students. J Exerc Rehabil. 2022;18:389–94.

161. Kurucz R, Fox E, Mathews D. Construction Of A Submaximal Cardiovascular Step Test. Research Quarterly. 1969;40:115–22.

162. Silva Magalhaes Mg, Teixeira Jb, Bezerra Santos Am, Silva Climaco Dc, Santos Silva Tn, Jaguaribe De Lima Am. Construct Validity And Reproducibility Of The Six-Minute Step Test In Subjects With Obstructive Sleep Apnea Treated With Continuous Positive Airway Pressure. J Bras Pneumol. 2020;46:E20180422.

163. Skranc O, Havel V, Bartak K. Comparison Of Work Capacity Measured By Graded Step-Test And On A Bicyle Ergometer. Ergonomics. 1970;13:675-+.

164. Aadahl M, Zacho M, Linneberg A, Thuesen Bh, Jorgensen T. Comparison Of The Danish Step Test And The Watt-Max Test For Estimation Of Maximal Oxygen Uptake: The Health2008 Study. Eur J Prev Cardiol. 2013;20:1088–94.

165. Pepera G, Panagiota Z. Comparison Of Heart Rate Response And Heart Rate Recovery After Step Test Among Smoker And Non-Smoker Athletes. Afr Health Sci. 2021;21:105–11.

166. Bellet S, Roman L. Comparison Of Double 2-Step Test And Maximal Exercise Treadmill Test - Studies In Coronary-Prone Subjects. Circulation. 1967;36:238-.

167. Silva De Andrade Ch, De Camargo Aa, De Castro Bp, Malaguti C, Dal Corso S. Comparison Of Cardiopulmonary Responses During 2 Incremental Step Tests In Subjects With Copd. Respir Care. 2012;57:1920–6.

168. Murayama M. Comparison Of Amount Of Multistage Bicycle Exercise Test To That Of Masters Two-Step Test. Jpn Circ J-Engl Ed. 1973;37:904–904.

169. Welch G, Bruce R, Bridges W, Johnson A, Lehmann J, Nielsen M. Comparison Of A New Step Test With A Treadmill Test For The Evaluation Of Cardiorespiratory Working Capacity. Am J Med Sci. 1952;223:607–17.

170. Bouisset S, Monod H. Comparison Of A Body Morphology Index (Pignet) And 2 Tests Of Physical-Fitness (Ruffier And Harvard Step-Test) - Morphological Versus Physiological Assessment Of Physical Working Capacity. Trav Hum. 1961;24:213–24.

171. Beaumont M, Losq A, Peran L, Berriet A-C, Couturaud F, Le Ber C, Et Al. Comparison Of 3-Minute Step Test (3mstept) And 6-Minute Walk Test (6mwt) In Patients With Copd. Copd-J Chronic Obstr Pulm Dis. 2019;16:266–71.

172. Shapiro A, Shvartz E, Magazanik A, Shapiro Y. Comparison Of 3 Step Tests For Prediction Of Maximal Oxygen-Consumption In Young Male Subjects. Isr J Med Sci. 1973;9:518–518.

173. Kamide H. Comparative Sensitivity Evaluation Of Masters Double 2 Step Test And Hypoxemia Test In Coronary Suspected Subjects. Jpn Circ J-Engl Ed. 1974;38:331–3.

174. Kamide H, Miyazawa T, Furuta K, Goto N, Higuchi H, Higashihara Y, Et Al. Comparative Evaluation On Electrocardiographic Changes Of Masters Double 2-Step Test And Levys Hypoxemia Test. Japanese Circulation Journal. 1969;33:864-+.

175. Master A, Rosenfel. I. Clofibrate And Master 2-Step Test. Circulation. 1969;40:Ii17-+.

176. Karloh M, Correa Ks, Martins Lq, Araujo Clp, Matte Dl, Mayer Af. Chester Step Test: Assessment Of Functional Capacity And Magnitude Of Cardiorespiratory Response In Patients With Copd And Healthy Subjects. Braz J Phys Ther. 2013;17:227–35.

177. Maggio Ab, Crettenand A, Vuistiner P, Tabin R, Praz M, Martin Xe, Et Al. Chester Step Test To Estimate Maximal Oxygen Consumption In Lean And Obese Children. Swiss Med Wkly. 2014;144:33s-33s.

178. De Camargo Aa, Justino T, Silva De Andrade Ch, Malaguti C, Dal Corso S. Chester Step Test In Patients With Copd: Reliability And Correlation With Pulmonary Function Test Results. Respir Care. 2011;56:995–1001.

179. Molloy Ms, Robertson Cm, Ciottone Gr. Chester Step Test As A Reliable, Reproducible Method Of Assessing Physical Fitness Of Disaster Deployment Personnel. Southmedj. 2017;110:494–6.

180. Lee Ht, Roh Hl, Kim Ys. Cardiorespiratory Endurance Evaluation Using Heart Rate Analysis During Ski Simulator Exercise And The Harvard Step Test In Elementary School Students. J Phys Ther Sci. 2016;28:641–5.

181. De Souza Yr, Maynard Da Silva K, Gomes J, Varella I, Figueira B, Da Cal M, Et Al. Cardiac Overload Evaluated By Doubled-Product Between The Six-Minutes Walk Test And Six-Minutes Step Test. Am J Respir Crit Care Med. 2015;191.

182. Da Costa Ch, Da Silva Km, Maiworm A, Raphael Y, Parnayba J, Da Cal M, Et Al. Can We Use The 6-Minute Step Test Instead Of The 6-Minute Walking Test? An Observational Study. Physiotherapy. 2017;103:48–52.

183. Petrella Rj, Koval Jj, Cunningham Da, Paterson Dh. Can Primary Care Doctors Prescribe Exercise To Improve Fitness? The Step Test Exercise Prescription (Step) Project. Am J Prev Med. 2003;24:316–22.

184. Master A, Rosenfel. I. Can Amount Of S-T Segment Depression After 2-Step Test By Correlated With Severity Of Ischemic Heart Disease. Am J Cardiol. 1965;15:139-.

185. Burt J, Blyth C, Rierson H. Body-Fat, Blood-Coagulation Time, And The Harvard Step Test Recovery Index. Res Q. 1962;33:339–42.

186. Vilarinho R, Serra L, Esteves C, Caneiras C, Montes Am. Between-Day Reliability Of A Novel Incremental Step Test To Assess Exercise Capacity In Copd Patients. Eur Resp J. 2021;58.

187. Master A. Augmented Master Double 2-Step Test. Circulation. 1973;48:249–249.

188. Fyffe A, Bogg T, Orr R, Browne Gj. Association Of Simple Step Test With Readiness For Exercise In Youth After Concussion. J Head Trauma Rehabil. 2020;35:E95–102.

189. So R, Murai F, Matsuo T. Association Of Cardiorespiratory Fitness With The Risk Factors Of Cardiovascular Disease: Evaluation Using The Japan Step Test From The National Institute Of Occupational Safety And Health. J Occup Health. 2022;64:E12353.

190. Bandyopadhyay B, Chattopadhyay H. Assessment Of Physical-Fitness Of Sedentary And Physically Active Male College-Students By A Modified Harvard Step Test. Ergonomics. 1981;24:15–20.

191. Gupta P, Kumar B, Banothu Kk, Jain V. Assessment Of Cardiorespiratory Fitness In 8-To-15-Year-Old Children With Overweight/Obesity By Three-Minute Step Test: Association With Degree Of Obesity, Blood Pressure, And Insulin Resistance. Indian J Pediatr. 2023;90:1216–22.

192. Master A, Rosenfel. I. Arrhythmias In 2-Step Test - Monitored And After Exercise. Circulation. 1967;36:Ii26-+.

193. Master A. Arrhythmias In 2-Step Test - 600 Patients. Circulation. 1971;44:22-.

194. Master A, Rosenfel. I. Arrhythmias In 2-Step Test. Circulation. 1966;34:I22-.

195. Hansen D, Jacobs N, Bex S, D’haene G, Dendale P, Claes N. Are Fixed-Rate Step Tests Medically Safe For Assessing Physical Fitness? Eur J Appl Physiol. 2011;111:2593–9.

196. Burge At, Rodrigues Jc, Abramson Mj, Cox Ns, Bondarenko J, Webb E, Et Al. Application Of The Modified Incremental Step Test For Pulmonary Rehabilitation. Phys Therapy. 2021;101:Pzab044.

197. Carvalho Idc, Ferreira Dk Da S. Applicability Of The Step Test For Physical Fitness Assessment Of Women With Chronic Venous Disease Symptoms: A Cross-Sectional Study. J Vasc Bras. 2022;21:E20220092.

198. Petrella Rj, Wight D. An Office-Based Instrument For Exercise Counseling And Prescription In Primary Care - The Step Test Exercise Prescription (Step). Arch Fam Med. 2000;9:339–44.

199. Wang K, Zhang T, Ouyang Y, Jiang H, Qu M, Peng L, Et Al. An Experimental Study Of Step Test Index Combined With Heart Rate Variability In Estimating Maximum Oxygen Uptake In Women With Drug Use Disorder. Front Physiol. 2020;11:322.

200. Culpepper M, Francis K. An Anatomical Model To Determine Step Height In Step Testing For Estimating Aerobic Capacity. J Theor Biol. 1987;129:1–8.

201. Maggio Abr, Vuistiner P, Crettenand A, Tabin R, Martin Xe, Beghetti M, Et Al. Adapting The “Chester Step Test” To Predict Peak Oxygen Uptake In Children. Swiss Med Wkly. 2017;147:W14435.

202. Vilarinho R, Mendes Ar, Gomes M, Ferreira R, Costa F, Machado M, Et Al. Adapted Chester Step Test Can Have Maximal Response Characteristics For The Assessment Of Exercise Capacity In Young Women. Healthcare. 2021;9:308.

203. Dal Corso S, De Camargo Aa, Izbicki M, Malaguti C, Nery Le. A Symptom-Limited Incremental Step Test Determines Maximum Physiological Responses In Patients With Chronic Obstructive Pulmonary Disease. Respir Med. 2013;107:1993–9.

204. Dal Corso S, Malaguti C, De Camargo Aa, Izbicki M, Nery Le. A Symptom-Limited Incremental Step Test Determines Maximal Physiological Responses In Patients With Copd. Eur Resp J. 2012;40.

205. Dal Corso S, Duarte Sr, Neder Ja, Malaguti C, De Fuccio Mb, De Castro Pereira Ca, Et Al. A Step Test To Assess Exercise-Related Oxygen Desaturation In Interstitial Lung Disease. Eur Resp J. 2007;29:330–6.

206. Pitetti Kh, Fernhall B, Stubbs N, Stadler Lv. A Step Test For Evaluating The Aerobic Fitness Of Children And Adolescents With Mental Retardation. Pediatr Exerc Sci. 1997;9:127–35.

207. Simsic Aa, Gastaldi Ac, Baddini-Martinez J. A Six-Minute Step Test Protocol For The Investigation Of Dyspnea. J Bras Pneumol. 2014;40:673–4.

208. Vieira E, Costa L, Amorim B, Oliveira L, Silva T, Sperandio P, Et Al. A Simplified Bode Score To Predict Mortality In Patients With Copd: The Role Of 4-Min Step Test. Eur Resp J. 2017;50.

209. Siconolfi S, Garber C, Lasater T, Carleton R. A Simple, Valid Step Test For Estimating Maximal Oxygen-Uptake In Epidemiologic Studies. Am J Epidemiol. 1985;121:382–90.

210. Francis K, Feinstein R. A Simple Height-Specific And Rate-Specific Step Test For Children. Southmedj. 1991;84:169–74.

211. Feinstein R, Francis K. A Simple Height And Rate Specific Step-Test For Children And Adolescents. Pediatr Res. 1990;27:A4–A4.

212. Petrella Rj, Koval Jj, Cunningham Da, Paterson Dh. A Self-Paced Step Test To Predict Aerobic Fitness In Older Adults In The Primary Care Clinic. J Am Geriatr Soc. 2001;49:632–8.

213. Astrand P, Ryhming I. A Nomogram For Calculation Of Aerobic Capacity (Physical Fitness) From Pulse Rate During Submaximal Work. J Appl Physiol. 1954;7:218–21.

214. Francis K. A New Single-Stage Step Test For The Clinical-Assessment Of Maximal Oxygen-Consumption. Phys Ther. 1990;70:734–8.

215. Shephard R. A New Progressive Step Test For Prediction Of Maximal Oxygen Comsumption. Federation Proceedings. 1966;25:797-.

216. Ashley Cd, Smith Jf, Reneau Pd. A Modified Step Test Based On A Function Of Subjects’ Stature. Percept Mot Skills. 1997;85:987–93.

217. Sloan A. A Modified Harvard Step Test For Women. J Appl Physiol. 1959;14:985–6.

218. Francis K, Brasher J. A Height-Adjusted Step Test For Predicting Maximal Oxygen-Consumption In Males. J Sports Med Phys Fit. 1992;32:282–7.

219. Selland Ca, Kelly J, Gums K, Meendering Jr, Vukovich M. A Generalized Equation For Prediction Of Vo _2_ Peak From A Step Test. Int J Sports Med. 2021;42:833–9.

220. Bell J, Bassey E. A Comparison Of The Relation Between Oxygen-Uptake And Heart-Rate During Different Styles Of Aerobic Dance And A Traditional Step Test In Women. Eur J Appl Physiol. 1994;68:20–4.

221. Skranc O, Bartak K, Havel V. A Comparison Of Load Using Step-Test And Ergometer. Physiologia Bohemoslovaca. 1969;18:513-.

222. Marley W, Linnerud A. 3-Year Study Of Astrand-Ryhming Step Test. Research Quarterly. 1976;47:211–7.

223. Tancredi G, Quattrucci S, Scalercio F, De Castro G, Zicari Am, Bonci E, Et Al. 3-Min Step Test And Treadmill Exercise For Evaluating Exercise-Induced Asthma. Eur Resp J. 2004;23:569–74.

224. Master A. 2-Step Test Results Correlated With Findings Years Later At Death. Circulation. 1973;48:250–250.

225. Master A, Rosenfel. I. 2-Step Test After Myocardial Infarction. Circulation. 1967;36:Ii26-+.
